# Advancements in Regenerative Hydrogels in Skin Wound Treatment: A Comprehensive Review

**DOI:** 10.3390/ijms25073849

**Published:** 2024-03-29

**Authors:** Gabriel Olteanu, Sorinel Marius Neacșu, Florin Alexandru Joița, Adina Magdalena Musuc, Elena Carmen Lupu, Corina-Bianca Ioniță-Mîndrican, Dumitru Lupuliasa, Magdalena Mititelu

**Affiliations:** 1 Department of Clinical Laboratory and Food Safety, Faculty of Pharmacy, Carol Davila University of Medicine and Pharmacy, 020956 Bucharest, Romania; gabriel.olteanu@mst.umfcd.ro (G.O.); magdalena.mititelu@umfcd.ro (M.M.); 2 Department of Pharmaceutical Technology and Bio-Pharmacy, Faculty of Pharmacy, Carol Davila University of Medicine and Pharmacy, 020945 Bucharest, Romania; sorinel-marius.neacsu@drd.umfcd.ro (S.M.N.); dumitru.lupuliasa@umfcd.ro (D.L.); 3 “Ilie Murgulescu” Institute of Physical Chemistry, 060021 Bucharest, Romania; 4 Department of Mathematics and Informatics, Faculty of Pharmacy, “Ovidius” University of Constanta, 900001 Constanta, Romania; clupu@univ-ovidius.ro; 5 Department of Toxicology, Faculty of Pharmacy, Carol Davila University of Medicine and Pharmacy, 020945 Bucharest, Romania; corina-bianca.ionita-mindrican@drd.umfcd.ro

**Keywords:** inflammatory processes, polymer hybrid hydrogels, wound healing process, regenerative medicine, protective barrier

## Abstract

This state-of-the-art review explores the emerging field of regenerative hydrogels and their profound impact on the treatment of skin wounds. Regenerative hydrogels, composed mainly of water-absorbing polymers, have garnered attention in wound healing, particularly for skin wounds. Their unique properties make them well suited for tissue regeneration. Notable benefits include excellent water retention, creating a crucially moist wound environment for optimal healing, and facilitating cell migration, and proliferation. Biocompatibility is a key feature, minimizing adverse reactions and promoting the natural healing process. Acting as a supportive scaffold for cell growth, hydrogels mimic the extracellular matrix, aiding the attachment and proliferation of cells like fibroblasts and keratinocytes. Engineered for controlled drug release, hydrogels enhance wound healing by promoting angiogenesis, reducing inflammation, and preventing infection. The demonstrated acceleration of the wound healing process, particularly beneficial for chronic or impaired healing wounds, adds to their appeal. Easy application and conformity to various wound shapes make hydrogels practical, including in irregular or challenging areas. Scar minimization through tissue regeneration is crucial, especially in cosmetic and functional regions. Hydrogels contribute to pain management by creating a protective barrier, reducing friction, and fostering a soothing environment. Some hydrogels, with inherent antimicrobial properties, aid in infection prevention, which is a crucial aspect of successful wound healing. Their flexibility and ability to conform to wound contours ensure optimal tissue contact, enhancing overall treatment effectiveness. In summary, regenerative hydrogels present a promising approach for improving skin wound healing outcomes across diverse clinical scenarios. This review provides a comprehensive analysis of the benefits, mechanisms, and challenges associated with the use of regenerative hydrogels in the treatment of skin wounds. In this review, the authors likely delve into the application of rational design principles to enhance the efficacy and performance of hydrogels in promoting wound healing. Through an exploration of various methodologies and approaches, this paper is poised to highlight how these principles have been instrumental in refining the design of hydrogels, potentially revolutionizing their therapeutic potential in addressing skin wounds. By synthesizing current knowledge and highlighting potential avenues for future research, this review aims to contribute to the advancement of regenerative medicine and ultimately improve clinical outcomes for patients with skin wounds.

## 1. Introduction

The skin, due to its position, has an important role in human health and an essential function in the protection of the body. Therefore, it is important to maintain its integrity. Any injury that affects the integrity of this vital organ represents a risk factor for the body and sometimes for its survival. The regeneration capacity of the skin is affected by numerous factors (age, physiological state, associated conditions, the severity of injury) and can be accelerated with the help of therapeutic formulas with local and systemic action. The use of dermato-cosmetic and therapeutic preparations based on hydrogels is often an effective method, accepted and tolerated very well by patients, with quick effects. Most problems are generally caused by chronic injuries, especially those that occupy large areas, some of which even put the patients’ lives at risk. Modern formulations aim to create a protective film that is resistant, compatible with the skin, and has the active ingredients. This will increase the therapeutic efficiency of the preparation by accelerating healing in the most effective form and in terms of the absence of signs with an unsightly effect. The introduction of hydrogel bases, including biodegradable and biocompatible polymer hybrid hydrogels, has resulted in remarkable achievements in regenerative therapies. These innovative materials represent a convergence of cutting-edge science and engineering principles, offering unparalleled versatility and efficacy in a variety of biomedical applications. By seamlessly integrating with biological tissues and providing a conducive environment for cellular proliferation and tissue regeneration, hydrogel-based therapies have demonstrated extraordinary potential in addressing numerous clinical challenges, particularly in the realm of wound healing. This paradigm shift towards advanced hydrogel formulations underscores a transformative approach towards achieving optimal patient outcomes and revolutionizing the landscape of regenerative medicine.

The skin, being the body’s largest organ, holds immense significance in maintaining overall health. Its crucial functions and role as a shield against various environmental elements, such as mechanical, chemical, and biological factors, emphasize the utmost importance of preserving its integrity [1]. It plays a vital role in the homeostasis and thermoregulation of the body, sensing the sensations of temperature, touch, and pressure, reducing the harmful effects of UV radiation, and strengthening the immune system to prevent infections [1]. Its self-healing capacity plays an important role in vitamin production [2,3].

**Figure 1:**
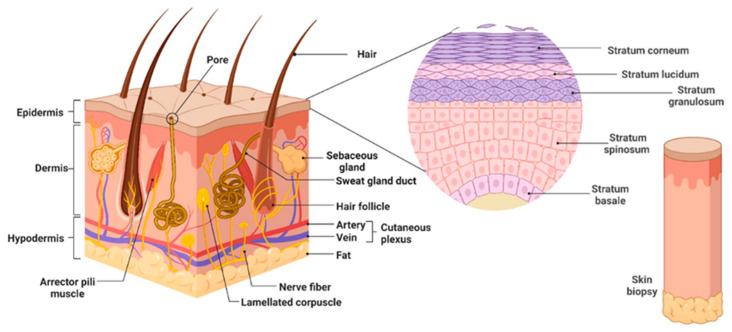
Skin anatomy. Created with BioRender.com (accessed on 15 October 2023).

Structurally, human skin consists of three distinct layers: the epidermis, dermis, and hypodermis (Figure 1). On the outside, there is an elastic layer (the epidermis) which is in the process of continuous regeneration. This layer consists of different cells (keratinocytes, corneocytes, melanocytes, Merkel cells, Langerhans cells) with the role of a protective barrier. Approximately 80% of the epidermal cells are represented by keratinocytes, which are constantly generated by division and gradually advance toward the surface of the skin. When they reach the surface, the cells flatten, die, shed, and are eliminated by natural exfoliation. The outer layer of the epidermis is relatively impermeable, keratinized, and corneous. At the level of the epidermis, melanin is produced by melanocytes. The pigment is involved in protection against ultraviolet radiation, Langerhans dendritic cells (approximately 2–8% of the total epidermal cells) are involved in immune defense, and Merkel cells (mechanoreceptors) have close connections with sensory neurons.

The epidermis which is not being vascularized receives nutrients by diffusion from the underlying connective tissue. The dermis houses nerve endings [2,4,5,6] that largely contribute to skin sensations, whereas touch sensations specifically stem from free nerve endings located in the epidermis.

There are four main transition zones and one transition zone at the level of the epidermis located from the inside to the outside: the stratum basale, stratum spinosum (30% of the thickness of the epidermis), stratum granulosum (10% of the thickness of the epidermis), stratum lucidum (a thin translucent area, the area of the transition between the granular and corneous layers), and stratum corneum [6].

There is an interface that acts as a separating membrane between the epidermis and dermis called the dermo-epidermal junction (DEJ), which is a specialized component of the extracellular matrix (ECM) and contains various proteins (laminin, nidogen, collagen type I, III, IV, VII, and XVIII proteoglycans, tenascin, and fibrillin-1) [6,7,8,9,10,11]. Fibronectin, a protein present in the dermo-epidermal junction, is the binder for basal keratinocytes [4,6] and is also involved in primary immunity. The DEJ is presented as a fibrous structure, plays an essential role in the regeneration process and cell migration, and has three layers (the lamina lucida, lamina densa, and lamina reticularis).

Under the epidermis is the thickest layer of the skin (up to 4 mm thick), the dermis, consisting of two layers: a layer rich in nerve endings and receptors and lymphatic and blood vessels (papillary dermis) and a layer in the form of a network of elastic fibers of collagen, elastin, and reticulin (synthesized by dermal fibroblasts) that form a support structure (reticular dermis), thus giving the skin properties of elasticity and mechanical resistance. At the level of the dermis, there is a rich network of nerves, blood vessels, and lymphatic vessels [4,6,12,13], as well as other types of cells such as human dermal microvascular endothelial cells (HDMECs), pericytes, and mast cells.

The deepest layer (the hypodermis), which accounts for approximately 20–25% of body weight in women and 15–20% in men, is considered an integral part of the subcutaneous adipose tissue.

The main component of the hypodermis is adipose tissue, which is accompanied by connective tissue and conjunctive vascular septa containing fibrocytes and mast cells. The functions of the hypodermis include the protection of the skin against musculoskeletal structures such as bones and muscles and thermal regulation, which helps to reduce heat loss [6,14,15].

Adipose tissue is white (WAT) or brown (BAT). The distinctive feature of brown adipose tissue (which occurs in adults in limited areas such as the axilla, neck, and perirenal area) consists of an increased density of mitochondria and cytochromes at the cell level, as well as a well-developed vascular network. It serves as an important and immediate source of heat production. Therefore, the hypodermis is recognized as an endocrine organ responsible for energy storage [1,6,16,17].

Physiologically, the skin has a slightly acidic pH that serves as a protective barrier against pathogen invasion and growth, and Langerhans cells in the epidermis are involved in fighting infections [14,18,19].

Despite the vast physiological involvement of the skin analyzer in the prevention of pathogen infections, there are situations where its extraordinarily laborious and complicated structure is compromised. The causes by which the skin can be compromised are various and involve mechanical, chemical, thermal, and electrical factors.

Regenerative hydrogels are a type of hydrogel designed specifically for applications in regenerative medicine and tissue engineering. These hydrogels possess unique characteristics that make them well suited for promoting tissue regeneration by promoting cell adhesion and growth. These hydrogels often contain bioactive components, such as growth factors, peptides, or other signaling molecules, to actively stimulate and support cellular activities crucial for tissue regeneration. Similar to conventional hydrogels, regenerative hydrogels are highly hydrophilic, enabling them to retain water and create a moist environment that supports cellular activities. Regenerative hydrogels, engineered to emulate the extracellular matrix (ECM) of tissues, exhibit a network of interlinked polymer chains adept at retaining significant water content without compromising structural stability. They provide a three-dimensional scaffold that mimics the natural ECM of tissues, offering support for cell attachment, migration, and tissue formation. Their composition renders them conducive to addressing skin lesions, primarily due to several critical factors. The mechanical properties of regenerative hydrogels can be tailored to match the mechanical characteristics of the target tissue. Firstly, their inherent biocompatibility ensures a non-reactive interface with living tissues, making them a viable choice for wound management applications. Many regenerative hydrogels are designed to be biodegradable, allowing them to degrade over time as new tissue forms. The degradation products should be non-toxic and easily metabolized by the body. Moreover, the hydrogels’ high water retention capability facilitates a moist microenvironment akin to the ideal conditions necessary for optimal wound healing. This moisture fosters crucial processes like cell migration, proliferation, and subsequent tissue regeneration. The ability to tailor the mechanical characteristics of hydrogels to resemble soft tissues is instrumental in providing a conducive milieu for cellular growth and tissue recovery. This adaptability allows for these materials to conform appropriately to wound contours, optimizing their therapeutic efficacy. Further enhancing their utility, hydrogels can be laden with therapeutic substances, including growth factors or pharmaceuticals, enabling controlled and sustained release directly at the wound site. This controlled delivery mechanism significantly bolsters cellular proliferation and tissue regrowth, expediting the healing process and providing a supportive environment for their survival and function.

Moreover, through structural modifications, hydrogels can be engineered to integrate cell-adhesive ligands, facilitating cell attachment, migration, and proliferation—vital processes in tissue regeneration. Additionally, their role as a protective barrier shield against external pathogens and environmental factors, mitigating infection risks and fostering an environment conducive to healing. Also, certain regenerative hydrogels are designed to promote the formation of new blood vessels (angiogenesis), a critical process for supplying nutrients and oxygen to regenerating tissues. In conclusion, the physical and chemical properties of regenerative hydrogels can be finely tuned to match the requirements of specific tissues or applications by adjusting parameters such as stiffness, porosity, and degradation rate.

In the realm of skin lesions, regenerative hydrogels serve as pivotal facilitators in the wound healing process by establishing an environment conducive to tissue regeneration. Their distinctive structural composition and versatile properties underscore their significance across diverse medical applications, spanning from advanced wound dressings to intricate tissue engineering endeavors, effectively supporting the body’s innate mechanisms for recuperation and repair.

In the present study, the characteristics of skin lesions or wounds, the dangers that may appear in skin lesions or wounds, the stages of regeneration processes, the characteristics and clinical applications of regenerative hydrogels, and the advantages of their use in the wound healing process are presented. Skin wound treatment has long been a challenge in healthcare, with traditional methods often falling short in promoting optimal healing and tissue regeneration. In this landscape of unmet clinical needs, the emergence of regenerative hydrogels presents a promising avenue for advancing wound care practices. These innovative biomaterials offer unique advantages, such as their ability to provide a moist wound environment conducive to healing, their tunable physical and chemical properties, and their potential to deliver bioactive agents to the wound site. However, despite their considerable potential, the full scope of benefits and applications of regenerative hydrogels in skin wound treatment remains to be fully elucidated. In this comprehensive review, we aim to address this gap by providing a thorough examination of the advancements in regenerative hydrogels and their specific benefits in skin wound treatment. By critically analyzing the latest research findings, discussing practical implications, and highlighting future directions, we aspire to offer valuable insights for researchers, clinicians, and healthcare practitioners seeking to optimize wound healing outcomes.

## 2. Characteristics of Skin Wounds

The characterization of skin wounds is a multifaceted process crucial for guiding effective treatment strategies tailored to individual patient needs. It entails a comprehensive assessment of diverse wound parameters to elucidate the underlying pathophysiology, assess healing progression, and identify potential complications. Central to this process is discerning the cause of the wound, whether it be traumatic, surgical, or related to underlying medical conditions such as diabetes or vascular insufficiency. The wound’s location on the body is also pivotal, as it influences healing dynamics and susceptibility to mechanical stress or infection. Moreover, the precise measurement of wound size and depth provides critical information about tissue loss and enables the accurate monitoring of the healing trajectory over time. The assessment of the wound bed’s appearance, including the presence of granulation tissue, necrosis, or slough, offers insights into tissue viability and the local microenvironment conducive to healing. Equally important is the evaluation of wound edges and the surrounding skin for signs of inflammation, maceration, or compromised perfusion, which may impact healing outcomes. The detection of infection, characterized by erythema, warmth, purulent exudate, and systemic symptoms, warrants prompt intervention to prevent further tissue damage and systemic spread. Additionally, categorizing wounds based on their healing stage (e.g., inflammatory, proliferative, remodeling) guides appropriate therapeutic interventions to facilitate progression through the healing continuum. Collectively, the thorough characterization of skin wounds serves as the cornerstone for formulating evidence-based treatment plans aimed at optimizing healing outcomes and promoting patient well-being. Wounds or skin lesions represent damage to the structural and functional integrity of tissues, including skin, mucous membranes, and organ tissues, and are a major risk for the spread of infectious agents [14,20,21]. The characteristics of skin wounds depend on factors such as the wound’s cause, size, depth, and the individual’s overall health. Understanding the characteristics of skin wounds is essential for appropriate wound management and treatment. Timely and proper care can contribute to optimal healing outcomes and minimize complications such as infection and scarring.

The Centers for Disease Control and Prevention (CDC) categorizes lesions into four primary classes (Table 1):

The characterization of skin wounds involves assessing several key factors critical for understanding the nature and progression of the injury. Firstly, identifying the cause of the wound, whether traumatic, surgical, or associated with underlying conditions, provides valuable insights into the healing process. The wound’s location on the body influences its susceptibility to mechanical stress and infection, guiding treatment decisions. The accurate measurement of size and depth is essential for monitoring healing progress and determining appropriate interventions. The evaluation of the wound bed’s appearance, along with the condition of its edges and surrounding skin, offers clues to tissue viability and the local wound environment. Detecting signs of infection is paramount for timely management and the prevention of complications. Additionally, categorizing wounds based on their healing stage aids in selecting optimal therapeutic approaches to promote effective wound closure. Overall, the comprehensive characterization of skin wounds enables clinicians to devise targeted treatment plans, ultimately enhancing patient outcomes and recovery.

Depending on the nature of the healing process, wounds can be divided into two main categories: acute wounds and chronic wounds.

**Figure 2:**
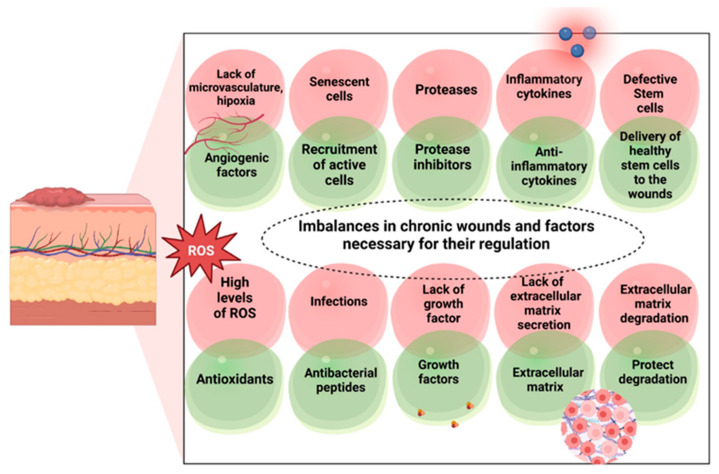
The imbalances present in chronic wounds (pink boxes) and the factors involved in combating them (green boxes) for regeneration. Created with BioRender.com (accessed on 15 October 2023).

Acute injuries are wounds that heal completely in about 8–12 weeks, leaving minimal scarring, and are mostly the result of mechanical injuries (rubbing the skin against hard surfaces such as knives or sharp edges) and surgical cuts. They can also be chemical injuries or burns caused by exposure to corrosive substances, radiation, electricity, or extreme temperatures (thermal injuries) [14]. In contrast, chronic wounds are characterized as wounds that do not normally heal in an orderly manner and within an adequate time frame. Typically, chronic wounds remain in the inflammatory phase of the healing process, with tissue repair delayed beyond 12 weeks from the initial time of trauma (Figure 2). As a result, chronic wounds attract high concentrations of pro-inflammatory cytokines, proteolytic enzymes (proteases), reactive oxygen species/free radicals (ROS), and senescent cells, which contribute to the maintenance of persistent infection and stem cell deficiency [23].

These wounds are mostly caused by repeated tissue insults or certain conditions, such as diabetes, the impairment of locoregional angiogenesis and innervation, or cell migration [24]. Other causes may be malignant tumors, infections, poor primary treatment, and other patient-dependent factors [25].

Keloid and hypertrophic scars represent an aberrant response to the normal wound healing process [26,27]. These scars are characterized by abnormal, irregular growth with excessive collagen formation and can be esthetically and functionally disruptive to patients. Although research in this field has aimed at identifying and approaching an optimal treatment for structural and functional tissue recovery, a unique, reliable, and effective treatment protocol for this type of scar is not yet known. However, it seems that surgical therapy consisting of excision followed by intralesional steroid injection in the postoperative period would have a reasonable outcome with a low recurrence rate. However, keloid and hypertrophic scars remain a challenge both for the medical staff who strive to apply an effective treatment and for the patient who has to continue their daily life with a diminished local functional capacity and low self-esteem due to esthetic changes [26,28].

Considered a major public health problem, burns are responsible for approximately 11 million annual casualties worldwide, resulting in 180,000 deaths, as well as significant morbidity, psychological trauma, and economic losses [29]. In Romania, the mortality rate due to burns is 4.41 times higher than that in the USA [30]. For example, between 2006 and 2015, there were 2666 deaths caused by burns, with an annual average of 267 [30]. In patients with burns, an excessive inflammatory state is maintained, there is an increase in the metabolic rate secondary to the acute inflammatory response with a persistence of up to 3 years, and there is a change in the physiology of blood coagulation through the imbalance of circulating proteins (protein C, protein S, antithrombin III, coagulation factor XIII, and coagulation factor VIIIa and Va) [31,32,33,34,35,36,37,38].

### The Healing Process of Skin Wounds

Following an injury to the skin, multiple organized reactions occur around the injured tissue, culminating in tissue healing. This complex and dynamic physiological phenomenon consists of four stages (three according to some authors, since hemostasis is considered to be part of the inflammatory phase), namely, hemostasis, the inflammatory phase, the proliferation phase (tissue growth), and the maturation and remodeling phase tissue [1,14,39,40].

**Figure 3:**
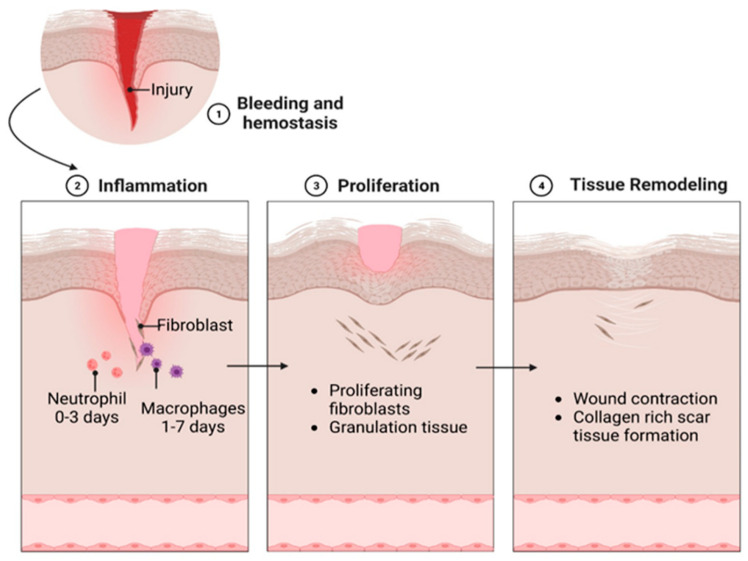
The main stages of the tissue regeneration process: inflammation, proliferation, and tissue remodeling. Created with BioRender.com (accessed on 23 October 2023).

Following an injury, a finely orchestrated sequence of actions is set in motion by receptors and cells that participate in the processes mentioned above. Each of these elements plays a pivotal role in advancing wound healing (Figure 3).

Thus, in the first minutes, vasoconstriction is performed to stop blood loss, and platelets adhere to the wound and subendothelial structures (collagen, basement membrane, and microfibrils). Later, platelet aggregation allows for platelets to join together with the production of membrane changes that lead to the grouping of IIb–IIIa complexes and the fixation of fibrinogen and calcium. Neutrophils and other inflammatory cells move to the wound site, causing the release of mediators and cytokines that play roles in angiogenesis, thrombosis, and re-epithelialization. In turn, fibroblasts stabilize the extracellular components that later play a supporting role [41,42].

In the inflammatory phase, neutrophils, monocytes, macrophages, lymphocytes, and other immune cells are recruited to the wound to phagocytose damaged or dead cells, bacteria, and other pathogenic microorganisms, as well as local debris [41,42,43]. The inflammatory phase lasts from 1 to 4 days post-injury and prepares the wound for regeneration through phagocytosis and waste removal [39,41,44,45].

After passing through the inflammatory phase, over a period of time between 5 and 20 days, there is the proliferation (proliferative phase) of vascular endothelial cells and fibroblasts at the wound level because of the secretion of growth factors by the inflammatory cells. After fibroblasts multiply, granulation tissue development becomes pivotal by generating extracellular matrix components such as proteoglycans, hyaluronic acid, procollagen, and elastin [41]. These compounds serve as an ideal base for the creation of new blood vessels (angiogenesis). The process of angiogenesis aims to supply oxygen and nutrients to cells [42]. The next stage of the proliferative or re-epithelialization phase is significant, namely, the migration of epithelial cells from the periphery of the wound/wound edges to the surface of the granulation tissue to cover the defect, a process also known as “re-epithelialization” [41,42,46,47].

The final stage of the healing process is the tissue maturation and remodeling phase, which begins approximately in the third week and can last up to 12 months. In this phase, the re-epithelialization and recovery of the tensile strength of the dermis occur. The resulting scar will be approximately 80% of its original strength. However, the scar will continue to remodel over a period of several months to several years [1,14,41,48].

In adults, the wound healing process is relatively slower than that in infants and often results in scarring (healing by fibrosis) rather than regeneration, further requiring innovative strategies for optimal and scar-free wound healing [49].

In general, age is one of the factors that influence the healing process. The ability to heal a wound is impaired by aging, leading to decreased skin strength and elasticity, decreased blood flow to the extremities, and psychological stress [41,50,51,52].

Other factors that alter the healing process include the sex of the individual, stress, bacterial colonization, reperfusion injuries, altered cellular response, collagen synthesis defects, obesity, smoking, alcoholism, vascular insufficiency, and certain pathologies such as diabetes, along with compromised nutritional or immunological status, which are major causes of non-healing skin wounds. Local factors that can influence the healing process include prolonged or repeated local pressure, hypoxia, ischemia, tissue edema, infections, maceration, and dehydration [41,50,51,52]. The healing process is dynamic and can vary based on the type and extent of the wound, as well as individual factors. Proper wound care, hygiene, and medical intervention, when necessary, can support natural healing mechanisms and contribute to optimal outcomes.

## 3. Applications of Hydrogels in Wound Regeneration

Hydrogels occupy a fundamental role in the landscape of regenerative medicine owing to their multifaceted advantages. Their propensity for biocompatibility allows for the emulation of the natural extracellular matrix, fostering an environment conducive to cellular growth, differentiation, and tissue regeneration. Notably, their high water content and porous structure closely mirror the native tissue milieu, facilitating crucial functions like nutrient exchange and cellular communication vital for tissue regeneration processes.

Functioning as carriers, hydrogels enable the controlled and localized delivery of growth factors, proteins, and therapeutic agents to specific sites, thereby augmenting tissue regeneration while mitigating systemic side effects. Acting as scaffolds, they offer mechanical support and spatial guidance, facilitating cellular proliferation and the assembly of new tissue, thereby assisting in the regeneration of compromised tissue.

The customizable properties of hydrogels, including stiffness, porosity, and degradation rates, afford adaptability to meet specific tissue requisites, optimizing the regenerative process across diverse applications. Certain formulations exhibit anti-inflammatory attributes, modulating immune responses to create a more conducive environment for tissue regeneration.

Their minimally invasive applicability in injectable, implantable, or topical forms enables precise localization at injury sites or tissue defects, enhancing targeted therapeutic interventions. In the domain of tissue engineering, hydrogels serve as foundational platforms facilitating cell attachment, growth, and differentiation, thereby contributing significantly to the creation of artificial tissues and organs.

Moreover, hydrogels exhibit versatility in supporting the regeneration of a broad spectrum of tissues, encompassing bone, cartilage, skin, neural, and cardiovascular tissues, catering to varied clinical demands. The controlled degradation rates of hydrogels allow for temporary structural support during tissue healing, gradually integrating with newly formed tissue and obviating the necessity for removal surgeries.

The amalgamation of these attributes distinctly positions hydrogels as versatile and promising tools within the realm of regenerative medicine. Their utilization offers compelling solutions for tissue repair, organ regeneration, and the advancement of therapeutic outcomes across diverse medical conditions and injuries.

### 3.1. Regenerative Hydrogels: Chemical Composition and Mechanisms of Action Involved in Wound Healing

Hydrogels, due to their specific structure and properties, have an extremely strong potential to become the future of regenerative medicine. Their properties of the mechanical, electrical, and chemical replication of the human skin give them an advantage over other types of dressings. They can be applied either topically or by spraying or in some selected cases, by injection (especially when it is necessary to administer some drugs/active substances).

The wound healing process is a complex one involving multiple factors (both internal and external), and the wound microenvironment significantly influences healing time. Therefore, in recent years, the use of advanced biomaterials has been intensively researched for therapeutic effects, especially anti-inflammatory ones, in the treatment of chronic wounds.

Ever since their discovery in the 1960s, synthetic hydrogels have been increasingly used in the engineering of biological systems [53].

Traditional/conventional wound dressings mainly focus on passively protecting wounds against external pollutants and invaders and cannot actively stimulate the wound healing process [54]. Traditional dressings are not effective in adequately treating chronic wounds, such as infections and wounds associated with diabetes. Therefore, it has become necessary to develop a new generation of dressings with functional properties that not only protect against physical injury but also accelerate the tissue regeneration process to facilitate wound healing [55,56].

Hydrogel-based dressings outshine conventional options such as bandages and gauze because of their remarkable characteristics, compatibility with bodily tissues, and exceptional ability to retain water. They maintain a moist environment for wounds and consistently absorb exudate [57]. Hydrogel-based dressings are also advantageous because of their ability to biodegrade, which prevents secondary deterioration during dressing replacement. This impressive capacity makes them ideal materials for wound care [58,59,60,61]. The ability to absorb water and swell in an aqueous environment is due to hydrophilic groups such as -NH2, -COOH, -OH, -CONH2, -CONH, and -SO3H [62,63,64,65,66,67,68,69].

In contrast to foams and films, hydrogels feature a three-dimensional porous structure akin to the natural extracellular matrix (ECM), offering an advantageous environment for cell growth and movement. These dressings can be custom-designed structurally and biochemically, allowing for diverse properties, with their anti-inflammatory function being widely used and prominent [55,70,71,72,73,74]. Over the past 60 years, hydrogels have been designed to be topically applied, injectable, and sprayed on many organs and tissues [75,76,77,78].

**Figure 4:**
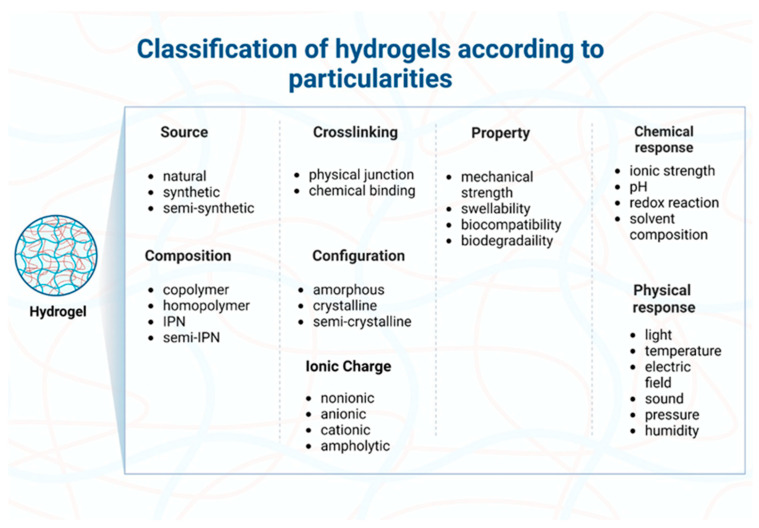
The main characteristics involved in the differentiation of healing hydrogels. Created with BioRender.com (accessed on 23 October 2023).

The categorization of hydrogels (illustrated in Figure 4) relies on various factors including their source, preparation methods, dimensions (macrogels, microgels, nanogels), composition (homopolymers, multipolymers or heteropolymers, copolymers [79], interpenetrating polymer networks, hybrids, composites), crosslinking, properties [80], responsiveness to environmental stimuli (physical, chemical, biochemical) [81,82], structure (amorphous or semicrystalline), degradation extent (biodegradable, bioabsorbable, bioerodible, controlled degradation) [83], and ionic charge (nonionic, ionic, zwitterionic, amphoteric) [84,85].

There are four types of natural biodegradable polymers used to make hydrogels used in clinical applications (the first two are from the polysaccharide category) [85,86,87,88,89,90,91,92]:

(1) Homopolysaccharides (cellulose and derivatives, pullulan and derivatives, dextran, gellan, carrageenans and sulfated derivatives, glycogen, inulin, gum guar, Acacia gum, pectin, and starch);
(2) Heteropolysaccharides (chitosan/chitin and derivatives, hyaluronic acid, chondroitin sulfate and other derivatives, xanthan gum, heparin, pectin, glycosaminoglycans/mucopolysaccharides, glucomannans, laminarin, proteoglycans, agar, gum arabic, gum tragacanth, and arabinoxylans);
(3) Polypeptides/proteins (gelatin, collagen, albumins, elastin, fibrin, fibronectin, fibrinogen, immunoglobulins, lactoferrin, casein, zein, soy protein, whey protein, calmodulin, prolamins (gluten and gliadin), protamines, lysozyme, histones, enzymes, hemoglobin, cytochrome C, and interferon);
(4) Polynucleotides and others (deoxyribonucleic acid/DNA and ribonucleic acid/RNA and lignin) [85,86,87,88,89,90,91,92].

The most used synthetic polymers for obtaining hybrid hydrogels can be classified into three main categories as follows: biodegradable, non-biodegradable, and bioactive polymers [85,93,94,95,96]. The most popular and widely used synthetic polymers are poly (ethylene glycol) (PEG), poly (lactic acid) PLA, copolymers, and poly (vinyl alcohol) (PVA) for the production of biodegradable hydrogels [85,97,98,99,100,101].

Hybrid polymer hydrogels are known for their biodegradability and biocompatibility, as well as the ability to allow for the passage of oxygen (increased permeability and sensitivity), nutrients, and water-soluble metabolites. They represent a promising option for cell encapsulation and show remarkable similarity to natural soft tissues. In the field of regenerative medicine, polymer hybrid hydrogels have demonstrated significant efficiency in tissue remodeling and the development of therapeutic delivery systems, facilitating cell attachment and proliferation [85,102,103]. By combining the properties of natural and synthetic polymers for the formation of hybrid hydrogels, an innovative approach with bioactive action in the field of tissue engineering is achieved. Thus, hybrid hydrogels are gaining popularity among researchers in this field who want to identify the best combinations and methods of administration for maximum efficacy.

### 3.2. The Role of Reactive Oxygen Species (ROS) from Regenerative Hydrogels in the Wound Healing Process

Reactive oxygen species (ROS) play a role in the field of regenerative hydrogels, particularly in the context of wound healing and tissue regeneration. In recent research, the focus has shifted toward understanding the role of reactive oxygen species (ROS), chemokines, and macrophage phenotypes as crucial elements contributing to heightened inflammation seen in skin or organ injuries [55,57,70,73,104,105]. Natural or synthetic polymers undergo physical rearrangement or chemical crosslinking to acquire diverse functions and properties. Crosslinking agents are used to form a three-dimensional network within the hydrogel. This network structure affects the hydrogel’s mechanical properties, stability, and drug release kinetics. Physically, hydrophobic associations, hydrogen bonds, and ionic interactions form, while chemically, polymers link via covalent bonds in chemical crosslinks, which may include disulfides, Schiff bases, and borate ester bonds. The methods of crosslinking vary based on the polymers’ nature [104,105].

**Figure 5:**
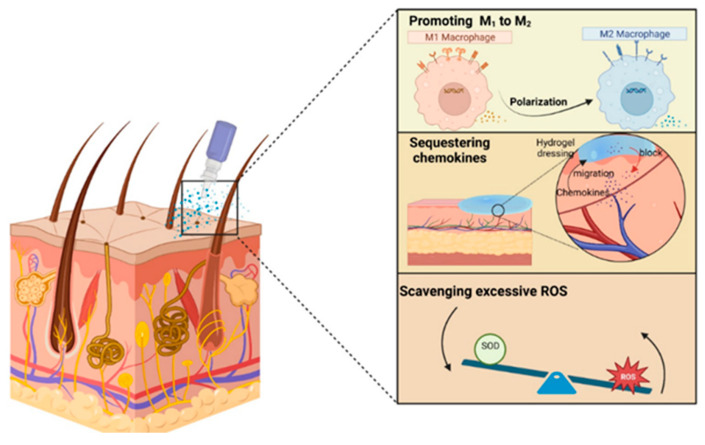
Action mechanisms of anti-inflammatory hydrogels on skin wounds. Created with BioRender.com (accessed on 23 October 2023).

Anti-inflammatory hydrogel dressings combine specific drugs, small bioactive molecules, and innovative biomaterials within a hydrogel matrix (as depicted in Figure 5). They can eliminate excess free radicals, sequester chemokines, and promote the polarization of M1-M2 macrophages, thus having the effect of reducing excessive inflammation in wounds and facilitating the healing process. These dressings act by stimulating angiogenesis (the formation of new blood vessels to improve locoregional vascularization), collagen deposition, epithelial cell migration, reduction in fibrosis (the exaggerated pathological development of connective tissues), and extracellular matrix remodeling [57].

Free radicals (ROS) are represented by hydroxyl radicals (OH), hydroxyl ions (OH-), superoxide anion (O^2−^), and peroxide (O_2_^2−^) [106,107]. Different studies have indicated that lower levels of reactive oxygen species (ROS) support the healing of tissues. Abnormal responses like heightened inflammation, seen in hypoglycemic conditions and infections, involve the substantial infiltration of inflammatory cells like neutrophils and macrophages, resulting in elevated ROS levels. This increase in ROS can have deleterious effects such as damage to DNA structures, membrane lipids and proteins, cell damage, and apoptosis [108,109,110,111,112,113].

### 3.3. Compounds with Antioxidant Properties used in Formulation of Regenerative Hydrogels for Wound Healing Process

It is known that an excess of ROS can have negative effects on the healing process. The use of compounds with antioxidant properties in hydrogels through various processes such as combination, modification, and polymerization has shown promising results in neutralizing excess ROS and promoting effective wound healing. Depending on their nature, these compounds have been classified into five categories as follows: (1) natural polyphenols, (2) polysaccharides, (3) amino acids, (4) synthetic polymers, and (5) novel metallic nanomaterials [114,115].

### 3.4. Natural Polyphenols

Phenolic hydroxyl groups from natural polyphenols have the ability to stabilize ROS through chemical modifications such as hydrogen shifts and electron transfer [116]. Moreover, natural polyphenols chelate transition metals and exert a protective and activating role on antioxidant enzymes by inhibiting oxidative enzymes resulting from oxidative stress but are also involved in antimicrobial protection [117,118,119]. Natural polyphenols are mainly compounds from the category of flavonoids (quercetin, catechin, catechol, curcumin) and acid ester polyphenols (ferulic acid, gallic acid, tannic acid, ester derivatives) [116,117,118,119,120,121,122,123,124,125].

For example, curcumin, the primary active component found in turmeric, possesses robust anti-infective, antioxidant, and anti-inflammatory attributes, making it a promising candidate for topical application on wounds [126]. In a study conducted by di Luca et al., a multifunctional compound was devised, merging curcumin-loaded hydrogels with microparticle systems incorporating polyphenols known for their antimicrobial properties and quercetin [124]. The final results demonstrated that the designed system reduced H_2_O_2_-induced oxidative cellular stress as well as the proliferation of methicillin-resistant Staphylococcus aureus. Another antioxidant bioactive compound is resveratrol (RSV), a polyphenol with excellent capacity for tissue regeneration, for modulating cytokine production, and insulin sensitivity [127]. Gallic acid, an important polyphenolic component, exhibits special properties, namely anti-inflammatory, antimicrobial, antibacterial, and ROS neutralization properties, thus considerably accelerating the wound healing process [122,128]. Ferulic acid is an organic phenolic compound derived from hydroxycinnamic acid, found in the cell wall of most plant species, linked to molecules such as arabinoxylans [129]. Wei et al. investigated the properties of ferulic acid by including it as a bioactive substance in a hydrogel with the aim of rapid wound healing [121]. The results were promising, as the antioxidant capacity of ferulic acid improved epithelial and connective tissue regeneration. Another natural polyphenol derived from plant species, widely used in bioengineering, is tannic acid. Hydrogels containing this bioactive compound, due to its valuable properties, show important characteristics such as adhesion, antibacterial activity, and an antioxidant effect. They lead to accelerated collagen deposition at the lesion and stimulate vascular endothelial growth factor (VEGF) expression while reducing tumor necrosis factor-alpha (TNF-α) levels [125,130].

### 3.5. Polysaccharides

Polysaccharides find wide applications across multiple medical domains like drug delivery, wound care, bioimaging, and tissue engineering. Thanks to their structure, which involves hydroxyl and carboxyl groups, polysaccharides possess the ability to participate in hydrogen and electron transfer reactions [131]. Importantly, polysaccharides exhibit significant antioxidant properties by directly or indirectly counteracting reactive oxygen species (ROS). They have the capacity to enhance the function of antioxidant enzymes while also impeding the activity of enzymes that promote oxidation. Thus, polysaccharides contribute to maintaining the redox balance in the body and protect against oxidative stress [131,132,133,134,135]. Among the polysaccharides with antioxidant effects that are incorporated into hydrogels are dextran, alginates extracted from seaweed, cellulose, chitin, chitosan, hyaluronic acid, and paramylon [136,137,138,139,140,141].

### 3.6. Amino Acids

Certain amino acids and peptides have the ability to directly interact with reactive oxygen species (ROS) via functional groups like amino (-NH_2_), hydroxyl (-OH), carboxyl (-COOH), and sulfur bonds. Specifically, amino acids that include hydroxyl or sulfhydryl (-SH) phenolic groups display a more notable antioxidant effect [142]. Among the amino acids incorporated in hydrogels that exert antioxidant and antibacterial effects are arginine, silk fibroin peptides, and pearl peptides [143,144,145,146,147,148,149].

It is important to note that the balance of ROS is critical, as excessive oxidative stress can lead to tissue damage. Regenerative hydrogels aim to harness the positive aspects of ROS while mitigating potential negative effects, contributing to the overall success of tissue regeneration and wound healing. The field of redox-responsive hydrogels continues to advance, offering innovative solutions for controlled and targeted therapeutic delivery in regenerative medicine.

All the natural materials mentioned above show good biocompatibility. However, one of their main disadvantages is increased enzymatic degradation as well as low physical and chemical stability. These aspects may limit their use in certain medical applications, as material durability and strength are critical factors for long-term therapeutic success [57]. However, these problems can be avoided or controlled by using synthetic materials, which will be discussed next.

### 3.7. Synthetic Polymers

In the field of synthetic polymers, research efforts have focused on the development of materials that compensate for the disadvantages of natural bioactive substances. Hydrogels containing synthetic polymers (polyvinyl alcohol/PVA, polyacrylic acid/PAA, polyamide polyesteramides/PEA, dopamine, puerarin) show high hygroscopic properties, antioxidant, antibacterial, and antimicrobial properties, and an acceleration of the wound healing process (cell proliferation, regeneration tissue, reducing the inflammatory phase of the healing process) [150,151,152,153,154,155].

### 3.8. Novel Metallic Nanomaterials

Recent studies have revealed that materials and their oxides can exhibit antioxidant properties when used in the form of nanomaterials. Specific metal oxide nanoparticles, like CeO_2_, selenium (Se), and Cu_5.4_O, demonstrate capabilities akin to antioxidant enzymes such as superoxide dismutase (SOD), catalase, and glutathione peroxidase [156,157,158]. Selenium nanoparticles (SeNPs) are acknowledged for their robust anticancer, antibacterial, antimicrobial, antiviral, anti-inflammatory, and antioxidant characteristics, all of which notably influence the process of wound healing. They are highly sought after due to their ability to support and accelerate tissue regeneration and promote effective wound healing [159,160,161,162,163].

CeO_2_ nanoparticles are currently used for a multitude of medical applications, drug therapy administration, and biosensing (biosensing, the detection of target molecules based on the principles used by a living system, such as the immune system) [57,164]. Hydrogels incorporating CeO_2_ nanoparticles are effective in wound healing due to antioxidant, antibacterial and antimicrobial effects [165].

Regarding Cu_5.4_O nanozymes, they were investigated for their ROS neutralization effect as well as in combination with heparin for chemokine sequestration. The hydrogel showed success in suppressing inflammatory cell migration (inflammatory chemokines such as monocyte chemoattractant protein-1 [MCP-1] and interleukin-8 [IL-8]) but also in neutralizing ROS from wound exudate with decreased oxidative stress cellular through the controlled release of Cu_5.4_O [166]. An intimate cellular mechanism that contributes to the acceleration of the wound healing process is the sequestration of chemokines, which leads to the reduction in local inflammation. Chemokines (chemotactic cytokines) are a superfamily of small proteins with a signaling role through G protein-coupled heptahelical chemokine receptors on the cell surface. They stimulate the directed movement of leukocytes (white cells) and endothelial and epithelial cells. Therefore, chemokines are of particular importance in the development and maintenance of the homeostasis of the immune system, being involved in all immune and inflammatory responses, whether protective or harmful in nature [167].

Chemokine receptors can bind multiple types of chemokines, while the same chemokine can interact with multiple types of receptors. This complex interaction between chemokines and receptors plays a fundamental role in the pathophysiological processes of chronic inflammation, tumorigenesis (tumor formation), and autoimmune diseases. Thus, chemokines are involved in the regulation and control of these pathological processes, having a significant impact on the functioning of the immune system and general health [168,169]. Being involved in all four phases of healing (hemostasis, the inflammatory phase, proliferation phase, and tissue maturation and remodeling phase), chemokines influence these phases through complex cellular mechanisms that determine angiogenesis, collagen deposition, and re-epithelialization, all of which have the same purpose, namely wound healing [170,171,172].

The persistence of chemokines and excessive infiltration into wound areas can have a negative impact on the wound healing process. Although chemokines are necessary in the initial stages of healing to recruit certain cells, an excessive and long-term presence can inhibit tissue regeneration and delay healing. This persistent infiltration can be associated with chronic inflammation and the formation of excess scar tissue, adversely affecting the healing process. Therefore, the regulation and balance of chemokine levels may play a crucial role in achieving efficient wound healing [173,174,175]. As a result of advanced research, effective therapeutic strategies have been identified to regulate the excessive proliferation of chemokines in wounds. These strategies include the use of monoclonal antibodies, small molecule antagonists, and glycosaminoglycans (GAGs) that have the ability to interfere with the distribution and activity of chemokines [170]. GAGs are negatively charged carbohydrate macromolecules (polysaccharides) that constitute important components of the ECM in connective tissues. GAGs (glycosaminoglycans) play roles in numerous pathological conditions like cardiovascular diseases, neurodegenerative diseases, and tumor processes [176,177].

In more complex wound scenarios, like diabetic wounds or burns, the local infiltration and persistence of inflammatory cells result in a significant release of pro-inflammatory chemokines such as MCP-1 and IL-8. These chemokines contribute to intensifying the invasion of inflammatory cells into the wound area, thus amplifying the cycle of chronic inflammation and perpetuating it over time [178,179].

It appears that GAG-based hydrogels with anti-inflammatory properties represent one of the leading therapeutic strategies for wound healing. They achieve beneficial interactions between GAGs and chemokines, thereby sequestering excess chemokines locally and ultimately contributing to the promotion of tissue healing [178,180,181].

It is important to highlight that the role of chemokines in the wound is extremely important and complex, and anti-inflammatory hydrogels are not aimed at sequestering or eliminating all chemokines but only those in excess such as MCP-1 and IL-8, thus promoting wound healing. Xu et al. fabricated a biomimetic hydrogel using polyvinyl alcohol (PVA) and chitosan (CS) as hybrid materials but loaded with chemotactic factor (SDF-1) for the rapid stimulation of hematogenous marrow mesenchymal stem cell (BMSC) recruitment in situ, for speeding up the tissue repair and regeneration process [182]. The loaded chemokines can be released in a controlled manner from the hydrogel and can recruit BMSCs both in vitro and in vivo. Incorporating specific chemokines that aid in wound healing into hydrogel dressings can significantly accelerate the overall healing timeline.

Macrophages participate in engulfing apoptotic neutrophils during the initial stages of healing, promoting angiogenesis, collagen deposition, and the migration of epithelial cells. Macrophages have been classified into pro-inflammatory macrophages (M1) and anti-inflammatory macrophages (M2), although this classification is a matter of debate. M1 macrophages produce ROS, nitric oxide (NO), interleukin-6 (IL-6), tumor necrosis factor-alpha (TNF-α), and matrix metalloproteinase 9, playing a crucial role in recognizing and removing pathogens, cellular debris, and apoptotic neutrophils during the early stages of the healing process. M2 macrophages support elevated levels of growth factors like platelet-derived growth factor (PDGF) and insulin-like growth factor-1 (IGF-1) while expressing arginase-1 (Arg-1), minimizing fibrosis and promoting collagen deposition, angiogenesis, and migration epithelial cells [183,184,185]. Some macrophages display both pro-inflammatory and anti-inflammatory phenotypes and may even exert other biological effects.

The phenomenon of macrophage polarization is strongly influenced by the specific microenvironment of the wound, which undergoes dynamic changes during the healing process, having a direct impact on the phenotype and functions of macrophages [186]. An urgent critical issue is that adverse factors such as hyperglycemia and bacterial infections prevent the transformation of pro-inflammatory (M1) macrophages into anti-inflammatory (M2) macrophages. As a result, the wound gets trapped in an inflammatory state, hindering epithelial regeneration, collagen formation, and angiogenesis. This obstacle prevents the shift toward the healing phase. Consequently, the sustained polarization of pro-inflammatory macrophages (M1) in wounds emerges as a critical concern demanding immediate attention [187].

Recent years have witnessed the development of various dressing types tailored to regulate the cellular environment (microenvironment) in chronic wounds. Their specific aim is to facilitate macrophage polarization during the latter stages of the healing process. One of the types of dressings that has attracted the most attention is represented by hydrogels. The use of hydrogels enables the immunomodulation of chronic wounds by releasing bioactive molecules, including antimicrobial substances, immunomodulatory components, growth factors, genes, and cells, which help promote the transition of macrophages from the M1 to M2 stage. Consequently, all of these accelerate the physiological process of tissue regeneration with wound healing [188,189,190,191,192,193,194,195].

Lactic acid-producing bacteria, often used as probiotics, have significant beneficial effects on protecting the host against harmful microorganisms, strengthening the host immune system, and reducing metabolic disorders [196]. In a study by Lu et al., a thermoresponsive hydrogel was formulated with heparin and poloxamer, which included a delivery method of live *Lactococcus bacteria* [197]. The lactic acid produced by these probiotic bacteria can cause macrophages to phenotypically transform into the M2 type, significantly favoring the angiogenesis process in diabetic wounds. Also, the resulting hydrogel can stimulate the production and protection of the growth factor VEGF, leading to the increased proliferation, migration, and tube formation of endothelial cells [197].

The specific physicochemical properties of any type of hybrid hydrogel are outstanding thermodynamic stability, high solubilization capacity [167], density, swelling/deflating capacity, high water content and permeability, low surface tension and low relative viscosity [175], rigidity, specific structure, sensitivity [127], biocompatibility and biodegradability (thus avoiding accumulation in organs) [195], non-immunological response, and the ability to undergo sterilization techniques, all of this of course alongside the elastic capacity and structural similarity to the ECM [48]. These properties can be improved by selecting the components that make up the hydrogel (the chemical composition, hydrophobic/hydrophilic ratio, and other complex biochemical interventions). Depending on their chemical processing to meet the needs of the clinical application, the degree of swelling/deflation, mechanical properties (a harder material is required for bone tissue compared to that used for adipose tissue), reactivity to external stimuli (the development of immunological reactions to contact with allergens from outside), and permeability can be modified [85,198].

**Figure 6:**
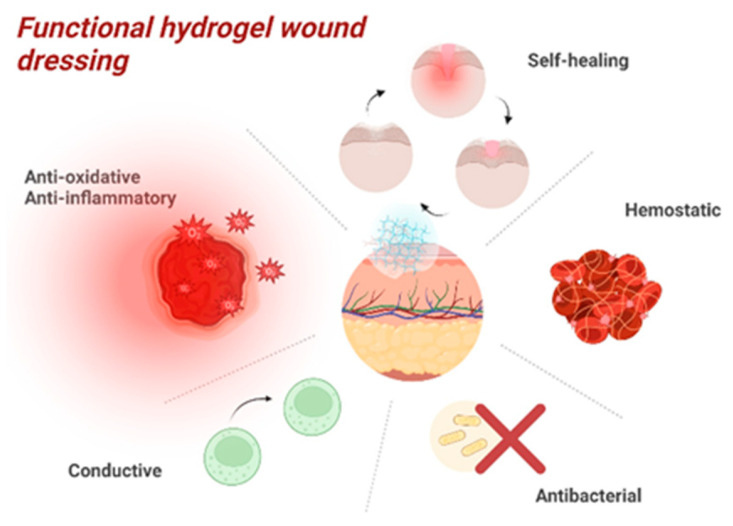
Important characteristics of a hydrogel-based dressing necessary for an effective regenerative therapeutic effect. Created with BioRender.com (accessed on 23 October 2023).

Figure 6 shows the functional properties of a hydrogel-based dressing, with properties necessary to accelerate the wound healing process without complications (bacterial infections, unsightly tissue retraction, excessive bleeding) [61].

### 3.9. Clinical Applications of Regenerative Hydrogels

Hydrogels possess the capacity to function as carriers for pharmaceutical agents, enabling the localized and controlled delivery of medications. These systems can be tailored to facilitate drug release triggered by specific stimuli such as variations in pH, temperature alterations, or enzymatic activity, thereby exhibiting suitability for targeted therapeutic interventions. Moreover, hydrogel-based dressings foster a conducive environment for wound recovery by ensuring the moisture retention of the wound bed. This aids in the facilitation of autolytic debridement and acts as a protective barrier against extraneous contaminants. Additionally, certain hydrogel formulations incorporate antimicrobial components, augmenting their utility in averting infections. Hydrogels have a three-dimensional structure and a network of hydrophilic polymers capable of absorbing water in addition to biological fluid [199,200,201,202,203,204,205,206,207]. Thus, they can build a soft and wet 3D structure, which is similar to that of the extracellular matrix, available to encapsulate the cells. This important aspect leads to those hydrogels enjoying increased popularity in application as wound healing dressings [208,209].

**Figure 7:**
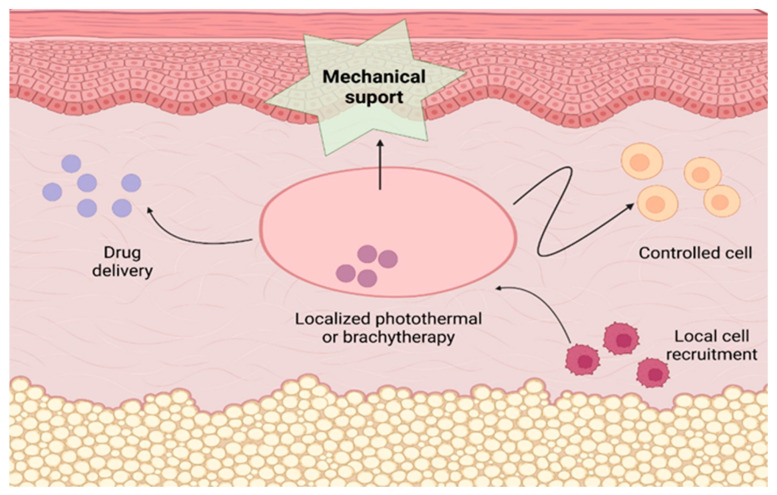
Therapeutic design strategies for regenerative hydrogels. Created with BioRender.com (accessed on 25 October 2023).

Hydrogels used for tissue regeneration are often designed to provide a physicochemical environment conducive to cell growth and differentiation and subsequent tissue regeneration (Figure 7). These modifications mainly consist of chemical regulation on the porosity, cell adhesion ligands, and viscoelasticity of the hydrogels [210,211,212,213].

The mechanical properties of the hydrogel significantly affect local cell recruitment and differentiation. For example, soft hydrogels have been shown to promote adipogenesis, while stiffer hydrogels induce stem cell osteogenesis [214,215]. Also, hydrogels are increasingly modified and designed to specifically recruit local immune cells and exert immunomodulatory effects, for example, for cancer therapy [216,217,218,219,220].

Hydrogels have several applications for treating imbalances or trauma that can lead to tissue damage, such as skin or organ damage. They can be used as mechanical support for the regeneration and healing of damaged tissues. In addition, hydrogels can be used for the controlled release of drugs or cells [221,222,223,224,225,226,227,228,229,230,231,232,233,234,235,236,237,238,239,240], local cell recruitment [216,217,218,219,221,241], or repeated localized radiotherapy [242,243], providing a precise and efficient way to deliver them to affected areas. This ability to deliver active substances or cells in a controlled manner to specific tissues makes hydrogels a promising option in regenerative medicine and therapy.

The characteristics of hydrogel-based dressings (a hemostatic effect, the stimulation of angiogenesis, and antibacterial, antimicrobial, and anti-inflammatory properties) make them ideal for wound healing, the prevention of subsequent infections, and the prevention of unsightly scar formation. Natural polymers, such as cellulose, chitosan, collagen, and hyaluronic acid, contain endogenous factors with a bioactivation role; therefore, they are exceptional substances for speeding up the healing process and maintaining a reduced microbial status at the wound level.

Ying H. et al. succeeded in adapting a hydrogel based on collagen and hyaluronic acid to promote spontaneous wound healing. At the same time, this hydrogel also exerted an antibacterial effect by inhibiting the growth of *Escherichia coli* and *Staphylococcus aureus* [244].

In addition to hemostatic and wound healing-enhancing effects, hydrogels are synthesized to exert antibacterial, antimicrobial, anti-inflammatory, and local adhesion effects at the wound level [244,245,246,247,248,249]. By combining these multiple effects, hydrogels can provide complete therapeutic support and facilitate effective wound healing.

Hydrogels can be used in addition to acute and chronic wound therapy, in more serious conditions such as myocardial infarction [250,251,252,253,254,255,256,257,258,259,260], spinal cord injury [261,262,263,264,265,266,267,268,269,270,271,272,273,274,275,276,277,278,279,280,281], intervertebral disc degeneration leading to low back pain (LBP) over time [282,283,284,285,286,287], and stroke [288,289,290,291,292,293,294], in the administration of neuroprotective therapies to counteract neurodegenerative diseases such as Parkinson’s disease and Huntington’s chorea (Huntington’s disease) [295,296,297,298,299], in the administration of therapy in oncological conditions [221,222,223,224,225,226,227,228,229,230,231,238,240,241,300,301,302,303,304,305,306,307], eye disorders [308,309,310,311,312,313,314,315,316,317,318,319,320,321,322], bone disorders, and/or bone regeneration [323,324,325,326,327,328,329], and in the prevention of postoperative adhesions or complications [330,331,332,333,334,335].

Clinical studies exploring the regenerative capacity of healing hydrogels have shown promising results across various applications. Table 2 summarizes the biological and therapeutic effects of certain hydrogels according to the bioactive substances in their composition, as well as by combining them with other substances to obtain a chemical balance that reduces the risks of administration. The table summarizes the composition of the hydrogels, the mode of administration, and the type of subject investigated in the study (laboratory animal). Clinical studies on different experimental animals have demonstrated the effectiveness of different regenerative hydrogels in accelerating the wound healing process, some of the hydrogels also have a significant anti-inflammatory or antimicrobial effect, stimulating collagen synthesis or even antioxidant effects determined by the composition of the hydrogels, as can be seen from Table 2.

Although clinical research has demonstrated potential, the efficacy of hydrogels can be contingent upon variables such as their composition, structural attributes, and particular therapeutic use. Further investigations are imperative to refine hydrogel compositions, dosage strategies, and delivery techniques to achieve optimal regenerative results across various injury types and tissue repair contexts. Moreover, extensive longitudinal studies are required to evaluate the long-term durability and enduring efficacy of regenerative therapies employing hydrogel-based interventions.

Self-healing hydrogels exhibit distinctive attributes that distinguish them within diverse applications. These hydrogels possess inherent capabilities for autonomous damage repair, manifesting the spontaneous restoration of their structural integrity upon compromise. Often reliant on dynamic crosslinking mechanisms, these gels feature reformable connections between polymer chains, facilitating structural reinstatement after post-breakage. Importantly, they demonstrate the capacity for enduring multiple healing cycles, offering successive repairs even after sustained damage events. Their robustness enables resilience against mechanical stress, ensuring the maintenance of structural integrity [352,353].

Furthermore, the adaptability of self-healing hydrogels allows for tailoring to specific applications, enabling adjustments in mechanical properties, responsiveness to stimuli, and healing proficiency. Some variants exhibit responsiveness to particular stimuli, such as alterations in temperature, pH, light exposure, or chemical cues, initiating the healing process upon the occurrence of damage. Notably, their biocompatibility renders them suitable for diverse biomedical applications, including drug delivery, tissue engineering, and wound healing [354].

Ongoing research focuses on environmentally sustainable formulations, integrating biodegradable or renewable materials to foster the development of more eco-conscious self-healing hydrogels. Their ability to prolong the lifespan of materials and devices by self-repair further underscores their significance, potentially extending the utility of various products [355,356].

The unique characteristics intrinsic to self-healing hydrogels serve as a catalyst for innovation across interdisciplinary domains like materials science, medicine, and engineering. This distinctiveness not only broadens their scope of application but also fosters the exploration and advancement of novel technologies.

Self-healing hydrogels exhibit substantial promise in an array of clinical applications, primarily attributable to their distinct properties. These hydrogels serve as versatile wound dressings, fostering a protective milieu while stimulating wound healing through autonomous repair mechanisms that restore the gel structure’s integrity. Moreover, their role as proficient carriers for controlled drug delivery systems augments targeted therapies by enabling the gradual release of medications or therapeutic agents, thereby mitigating systemic side effects [357].

In the realm of tissue engineering, self-healing hydrogels assume a pivotal role as scaffolds facilitating tissue growth and regeneration. Their ability to emulate the extracellular matrix enhances tissue repair and regeneration, particularly in applications concerning cartilage, bone, and other tissue engineering pursuits. Additionally, their utility extends to serving as biocompatible surgical adhesives, adept at sealing tissues and providing dynamic support during surgical procedures while retaining self-healing capabilities.

These hydrogels find utility in soft robotics and biomedical devices owing to their inherent capacity for damage repair, ensuring sustained functionality and durability within such applications. Explorations in ophthalmology demonstrate the potential for applications like contact lenses and ocular drug delivery systems, leveraging the self-healing attributes of these hydrogels for prolonged usability and enhanced comfort [358].

Their compatibility with neural tissues makes self-healing hydrogels promising in neural interface applications, offering the prospect of mitigating implant-induced damage and improving long-term biocompatibility and performance. Moreover, ongoing research endeavors focus on harnessing these hydrogels for orthopedic implants, aiming to enhance the longevity and functionality of such implants within the body [359].

Overall, the inherent self-healing properties of these hydrogels significantly augment the efficacy, durability, and safety of diverse clinical interventions across multifaceted medical disciplines. Ongoing research endeavors continue to explore and optimize their application in varied clinical settings, promising advancements and innovations in healthcare practices.

The constituents and arrangement of self-healing hydrogels demonstrate variability contingent upon the targeted application and desired functionalities. The essential elements and methodologies consistently employed in their formulation encompass distinct components [360,361]:

*Polymeric matrix*: Self-healing hydrogels predominantly rely on polymers as their fundamental matrix. Natural polymers such as alginate, chitosan, and collagen, alongside synthetic counterparts such as polyethylene glycol (PEG), polyvinyl alcohol (PVA), and polyacrylamide, define the hydrogel’s structure, porosity, and mechanical attributes.

*Crosslinking mechanisms*: Crosslinking agents play a pivotal role in establishing a network within the polymer matrix, bestowing stability and coherence upon the hydrogel. Chemical agents like glutaraldehyde or physical crosslinkers responsive to temperature, pH alterations, or light stimuli are commonplace in this regard.

*Self-healing attributes*: Integrating self-healing agents within the hydrogel formulation is critical for conferring its reparative properties. These agents encompass diverse mechanisms such as reversible covalent bonds (e.g., disulfide bonds), host–guest interactions (like cyclodextrin-encapsulated guest molecules), and physical interactions (e.g., hydrogen bonding or π-π stacking).

*Solvent medium*: Hydrogels are typically conceived in aqueous solutions or solvents to maintain their heightened water content, an imperative aspect for emulating the native tissue environment and fostering cellular proliferation.

*Supplemental additives*: Tailoring the hydrogel often involves the inclusion of additives designed to impart specific functionalities. For biomedical applications, the integration of bioactive components like growth factors, drugs, or nanoparticles facilitates functionalities such as targeted drug delivery or augmented tissue regeneration.

*Catalysts or initiators*: In instances where hydrogel formation necessitates specific reactions, catalysts or initiators serve as catalysts to instigate the crosslinking process, notably in procedures like photo-polymerization.

The fabrication techniques employed for self-healing hydrogels encompass a spectrum of methodologies, spanning physical blending, chemical reactions, and advanced methodologies like 3D printing, offering precise control over the hydrogel’s structure and attributes [362].

The meticulous customization of the formulation and composition remains pivotal to attain the desired mechanical robustness, biocompatibility, responsiveness to external stimuli, and self-repair capabilities aligned with the envisioned clinical or biomedical utility. The optimization of these constituents and their proportional ratios is a crucial determinant for achieving the targeted performance benchmarks of the self-healing hydrogel.

Self-healing hydrogels have demonstrated various applications in therapeutic domains [360,362,363,364,365]:

*Wound healing dressings*: Advanced wound dressings formulated with self-healing hydrogels showcase autonomous repair capabilities. These dressings create a favorable milieu for wound healing by fostering tissue regeneration while concurrently serving as a protective barrier against infections.

*Drug delivery systems*: The utilization of self-healing hydrogels as carriers for controlled drug delivery is notable. Their integration with therapeutic agents within the hydrogel matrix allows for precise and sustained release, amplifying therapeutic efficacy while mitigating systemic side effects.

*Tissue engineering scaffolds*: Within tissue engineering paradigms, self-healing hydrogels serve as pivotal scaffolds for tissue growth and regeneration. Offering mechanical support and an environment conducive to cell proliferation, they facilitate the restoration of damaged tissues, including cartilage, bone, and dermal structures.

*Biomedical implants and devices*: The integration of self-healing hydrogels into biomedical devices and implants capitalizes on their reparative capacity, ensuring device longevity and functionality. Their incorporation in orthopedic implants or neural interfaces bolsters biocompatibility and durability.

*Ophthalmic applications*: Self-healing hydrogels find utility in ophthalmology, particularly in innovations like contact lenses or ocular drug delivery systems. Their innate ability for self-repair extends wearability and augments comfort for patients.

These instances underscore the manifold therapeutic utility of self-healing hydrogels across diverse medical domains. Their deployment substantiates enhancements in treatment methodologies, the facilitation of tissue restoration, and the augmentation of therapeutic interventions’ durability and efficacy.

When natural sources are used to extract gelling substances (alginate, chitosan, gums, etc.), the quality of the raw material and the absence of toxic contaminants (heavy metals, pesticides, microplastics, etc.) are crucial [366,367,368,369,370,371].

Because of their specific structure and properties, hydrogels have an extremely strong potential to become the future of regenerative medicine. Traumatic injuries of any nature (mechanical, thermal, chemical, electrical) impose stress at the cellular level that can often unbalance the normal functioning of the biochemical and physiological healing processes of the skin lesions. Thus, a deficient healing process can lead to an abnormal closure of the wound, infection with pathogenic agents, and other aspects that attract problems of an esthetic nature but especially of a functional nature, because of the problem of affecting the activities of everyday life [372,373].

However, we cannot assess with certainty the safety of applying these hydrogels to the general population because most studies have been conducted on laboratory animals that have physiology distinct from that of humans. It is necessary to conduct some protocols and studies on the efficiency and safety of the application of hydrogels to accelerate the healing process in human subjects because there is a risk of triggering certain exaggerated responses of the immune system, such as allergies or even autoimmune diseases.

Creating a high-performance dressing requires a great deal of research work, well-developed protocols, the evaluation of the safety of administration and use in human subjects, and the detection of the most effective method of the application according to the type of wound, the stage of healing the wound is in, and analyzing the factors that can influence the healing process, all this combined of course with creativity.

Several hydrogel-based products have been commercialized and are available in the market. (i) Many soft contact lenses are made from hydrogel materials. These lenses are designed to provide comfort by retaining moisture and conforming to the shape of the eye. (ii) Hydrogel dressings are widely used for wound care. They create a moist environment to promote wound healing and may contain additives such as antimicrobial agents or growth factors. (iii) Hydrogel-based drug delivery systems are utilized for various pharmaceutical applications. These may include topical drug delivery, oral formulations, and injectable systems. (iv) Some implants in the medical field, such as those used for tissue augmentation or as supports in surgery, are made from hydrogel materials. In Table 3, some hydrogels currently used in the market are summarized.

The development and future perspectives concerning hydrogel-based dressings with multiple functions in wound monitoring and treatment purposes can be summarized as follows:

(i) Progress in multifunctional hydrogel-based dressings is the next step in future research. The design of hydrogel dressings to display and monitor the microenvironment of the wound with outstanding anti-inflammatory, mechanical, antibacterial, injectable antifeeding, and self-healing properties must be advanced.
(ii) The requirement in the design/synthesis of hydrogel-based dressings with complete involvement in the whole complex wound healing process is imperative and represents a pathway for further research in the clinical domain.
(iii) A great awareness to advance a hydrogel wound dressing that can simultaneously display the microenvironment of wounds and treatment functions may be the future tendency.

In Table 4, some hydrogels used in clinical trials on humans and their therapeutical effects are summarized and also the possible advantages and disadvantages.

## 4. Techniques for Improving the Bioavailability of Active Ingredients from Healing Hydrogels

**Figure 8:**
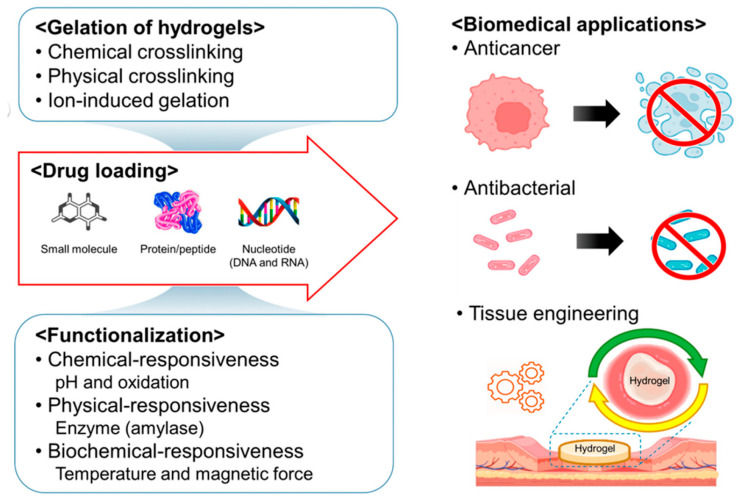
Exemplification of mechanisms available for improving bioavailability of active ingredients from hydrogels. Adapted from Ref. [395].

Improving the bioavailability of active ingredients from healing hydrogels involves optimizing the delivery system to enhance the absorption and efficacy of the therapeutic compounds [390,391,392,393,394]. The process is crucial for enhancing therapeutic outcomes. Bioavailability refers to the extent and rate at which a substance (e.g., a drug or bioactive compound) is absorbed and becomes available at the site of action (Figure 8). There are several techniques aimed at enhancing the bioavailability of active ingredients from healing hydrogels:

### 4.1. Nanoencapsulation

Incorporating active ingredients in nano-sized carriers within the hydrogel structure is a sophisticated approach that enhances drug delivery efficiency, controlled release, and the overall therapeutic impact. This technique, known as nanoencapsulation, involves encapsulating active compounds within nanoscale carriers, such as liposomes, micelles, or nanoparticles, and embedding them in a hydrogel matrix, protecting them from degradation and improving their stability [396,397]. The process of incorporating nanoencapsulated active ingredients in hydrogels offers some benefits: (i) it enhances the solubility and bioavailability of poorly water-soluble active ingredients, promoting better absorption; (ii) it protects the sensitive substances from external factors; (iii) it enables the controlled and sustained release of active ingredients, preventing rapid diffusion and ensuring a prolonged therapeutic effect; (iv) the hydrogel matrix allows for targeted delivery to specific tissues or cells, offering localized treatment and minimizing systemic side effects; and (v) it can accommodate various types of payloads, including drugs, genes, or imaging agents, providing versatility and allowing for synergistic therapeutic applications.

To encapsulate therapeutic compounds in nanocarriers within a hydrogel structure, different techniques are employed.

- Nanoprecipitation which involves the rapid mixing of a drug solution with a non-solvent in the presence of a stabilizer.
- Emulsion-solvent evaporation in which a solution of the active substance is emulsified in a water-immiscible solvent. The solvent is then evaporated, leaving behind nanoparticles.
- Emulsion-solvent diffusion, which is similar to emulsion-solvent evaporation but involves a slow diffusion process, resulting in smaller nanoparticles and more controlled release.
- Layer-by-layer assembly which involves the sequential adsorption of layers onto the hydrogel surface layer-by-layer, incorporating nanoparticles or active substances for controlled release.
- Electrospraying which can be used to create nanofibrous structures within the hydrogel, encapsulating nanoparticles and improving the surface area for drug release.

These methods are commonly used for the formation of nanoparticles, and when combined with hydrogel matrices, they enhance drug delivery efficiency and controlled release [398,399]. Incorporating active ingredients in nano-sized carriers within hydrogel structures is a powerful strategy for optimizing drug delivery systems. This approach offers numerous advantages, from enhanced stability and controlled release to improved bioavailability and targeted delivery, making it a promising avenue for advanced and effective therapeutic interventions.

### 4.2. Chemical Modification

The chemical modification of hydrogels involves altering their chemical structure to enhance specific properties such as mechanical strength, biocompatibility, responsiveness, or the ability to carry and release active compounds. This modification can be achieved through various chemical processes, and the choice of method depends on the desired properties and applications of the hydrogel [400,401]. The most common techniques used for the chemical modification of hydrogels are as follows:

- Crosslinking which involves the creation of covalent bonds between polymer chains within the hydrogel structure. Crosslinking enhances the mechanical strength, stability, and durability of hydrogels;
- Functional group modification based on the alteration of the functional groups of the hydrogel polymers to introduce desired properties. Functional group modification can enhance biocompatibility, promote cell adhesion, or facilitate the attachment of bioactive molecules;
- Surface modification which can modify the surface properties of the hydrogel to control interactions with biological entities. Surface modification can improve cell adhesion, protein adsorption, and bioactivity;
- Hydrogel copolymerization which combines different monomers to form copolymers with varied properties. Copolymerization allows for the tailoring of hydrogel properties, such as the swelling behavior, responsiveness to stimuli, or mechanical strength;
- The incorporation of responsive elements or stimuli-sensitive groups into the hydrogel structure. Responsive hydrogels can change properties (swelling, degradation, etc.), in response to environmental stimuli, such as pH, temperature, or light.

Chemical modification allows for precise control over the properties of hydrogels, making them versatile materials with applications across various fields, including drug delivery, tissue engineering, and biosensors.

### 4.3. pH and Temperature Responsiveness

The pH and temperature responsiveness of hydrogels are valuable properties that can be exploited for various applications, including drug delivery, tissue engineering, and diagnostics. pH and temperature-sensitive hydrogels undergo reversible changes in their physical or chemical properties in response to alterations in the surrounding pH or temperature. Both pH and temperature responsiveness contribute to the adaptability and versatility of hydrogels in the field of biomedicine, providing a platform for designing advanced drug delivery systems and tissue engineering constructs [402].

Understanding and tailoring the pH and temperature responsiveness of hydrogels enable precise control over their swelling behavior, stability, and drug release kinetics. These properties are crucial for applications where environmental responsiveness is desired, such as in drug delivery systems or smart biomaterials.

### 4.4. Incorporation of Penetration Enhancers

The incorporation of penetration enhancers in hydrogels is a strategy aimed at improving the permeation of active substances through biological barriers, such as the skin or mucous membranes. Penetration enhancers, also known as permeation enhancers or absorption enhancers, can facilitate the transport of drugs or therapeutic agents across these barriers. There are some key aspects and considerations for incorporating penetration enhancers into hydrogels:

(i) Chemical penetration enhancers: compounds that alter the physicochemical properties of the barrier, increasing the permeability, such as surfactants, fatty acids, and certain alcohols.
(ii) Physical penetration enhancers: methods that physically disrupt or modify the barrier, enhancing the transport of substances. These techniques include iontophoresis, sonophoresis, and microneedle application.

Incorporating penetration enhancers in hydrogels requires a careful balance between optimizing permeation and ensuring safety and biocompatibility. It is crucial to tailor the choice of enhancers and their concentration based on the specific requirements of the intended application and the nature of the active ingredients being delivered [403]. Incorporating penetration enhancers into responsive hydrogels offers a promising avenue for improving drug delivery efficiency, especially in clinical applications where controlled and targeted release is crucial for therapeutic success.

### 4.5. Hybrid Hydrogels

Hybrid hydrogels refer to materials that combine the characteristics of two or more different components, resulting in a synergistic system that exhibits unique properties. These hybrid structures often involve the integration of diverse materials, such as polymers, nanoparticles, or biological components. Hybrid hydrogels have gained considerable attention in various fields due to their versatility and the ability to tailor their properties for specific applications and therapeutic requirements. There are three types of hybrid hydrogels:

(i) Polymeric hybrid hydrogels that combine different types of polymers with complementary properties able to create a hybrid matrix with improved mechanical strength, stability, or responsiveness.
(ii) Nanocomposite hybrid hydrogels that integrate nanoparticles, such as nanoparticles of metals, metal oxides, or carbon-based materials, into the hydrogel matrix to enhance specific functionalities.
(iii) Biological hybrid hydrogels that combine hydrogels with biological components, such as proteins, peptides, or cells, to create biomimetic or bioresponsive materials.

Hybrid hydrogels provide a platform for designing advanced materials with tailored properties, making them suitable for a wide range of applications in medicine, environmental science, and materials science. The diversity of hybrid hydrogel systems allows for creative solutions to address specific challenges in various fields [404,405].

### 4.6. Surface Modification

The surface modification of responsive hydrogels is a crucial step to tailor their interactions with biological systems, enhance functionality, and optimize performance for specific applications. Surface modification can impart properties such as improved biocompatibility, controlled drug release, or responsiveness to external stimuli. The surface modification of hydrogels involves altering the outer layer of the hydrogel to impart specific properties, such as enhanced biocompatibility, controlled release, or improved interaction with biological entities. This modification can be achieved through various techniques (chemical grafting, physical adsorption, layer-by-layer assembly, plasma treatment) and can serve diverse applications like surface grafting or coating to modify the surface properties of hydrogel particles. The surface modification of hydrogels provides a versatile approach to tailoring their properties for specific applications, making them more functional and adaptable in diverse fields. The choice of modification technique and molecules depends on the desired functionality and the intended use of the hydrogel [406,407].

When implementing these techniques, it is important to consider the specific characteristics of the active ingredients, the intended therapeutic application, and the physiological environment in which the hydrogel will be used. Conducting thorough in vitro and in vivo studies can help validate the effectiveness of these strategies in improving bioavailability.

## 5. Conclusions

Regenerative hydrogels present significant advantages in wound healing and demonstrate compelling future prospects in healthcare. They foster an optimal microenvironment by preserving moisture, thereby facilitating cell migration, proliferation, and tissue regeneration, effectively mirroring the natural healing process. Their adaptability for customization enables tailoring to various wound types, sizes, and stages, accommodating diverse clinical requirements, whether addressing acute injuries, chronic wounds, or specific tissue demands. Moreover, their amenability to minimally invasive application techniques, such as injectable or topical forms, ensures precise wound site targeting, reducing patient discomfort and optimizing treatment precision. The incorporation of bioactive agents within these hydrogels facilitates controlled and localized drug delivery, thereby attenuating inflammation, fostering tissue regeneration, and preventing infections. Biocompatible formulations play a pivotal role in minimizing adverse reactions and scar formation at wound sites, culminating in improved healing outcomes and enhanced cosmesis. Functioning as scaffolds, these hydrogels facilitate tissue regeneration by mimicking extracellular matrix components, aiding in the regeneration of distinct tissues such as skin, cartilage, or internal organs.

Future avenues involve advancing biomaterials to develop hydrogels with heightened mechanical robustness, enhanced bioactivity, and responsive properties, thereby optimizing therapeutic efficacy. The integration of nanotechnology and smart materials into hydrogel formulations holds promise for real-time wound healing monitoring, responsive drug release, and tailored therapeutic interventions. Furthermore, synergistic amalgamations of hydrogels with adjunctive therapies, encompassing growth factors, stem cells, or bioactive molecules, stand poised to augment healing outcomes and expedite tissue regeneration. Research endeavors directing attention towards bioengineered skin substitutes utilizing hydrogels aim to revolutionize wound care by crafting functional equivalents for severe burns or chronic wounds. Anticipated progress in the clinical translation and commercialization of regenerative hydrogels holds the potential for transforming wound care practices, potentially yielding significant improvements in patient outcomes. The continued exploration of regenerative hydrogels in wound healing heralds promise for pioneering therapies, personalized medical approaches, and the evolution of advanced strategies in wound care.

## 6. Materials and Methods

We gratefully acknowledge the generous contributions from various sources that facilitated the execution of this study. To review the literature regarding the possible applications of hydrogels, especially their implication in accelerating the physiological process of wound healing, we performed a detailed search in the National Library of Medicine, Science Direct, Wiley Online Library, and Semantic Scholar, using specific keywords such as: ‘hydrogels’, ‘biomaterials’, ‘tissue engineering’, and ‘injectable hydrogels’. The search algorithm was then completed by using the terms ‘for wound healing’, ‘in chronic wounds’, and ‘for regenerative medicine applications’. After searching and filtering the articles, we included 406 bibliographic references from the following: NCBI—185 (of which, the most cited journals are as follows: *ACS Applied Materials & Interfaces*—9, *Advances in Wound Care (New Rochelle)*—7, *International Journal of Molecular Sciences*—7, *Biomacromolecules*—5, *Gels*—11, *Journal of Materials Chemistry B*—5, and *Molecules*—6), Science Direct—120 (*Biomaterials*—21, *Acta Biomaterialia*—14, *Carbohydrate Polymers*—11, *International Journal of Biological Macromolecules*—8, *Materials Science and Engineering: C*—7, and *Bioactive Materials*—6), Wiley Online Library—39 (*Advanced Materials*—6, *Advanced Functional Materials*—5, *Advanced Healthcare Materials*—5, and *Journal of Biomedical Materials Research*—4), and Semantic Scholar—7. We extend our sincere appreciation to all mentioned databases for providing the cited references essential for the successful completion of this study.

## Figures and Tables

**Table 1:** Wound classification according to CDC criteria [21,22].

Injury Classification Grades (I–IV)		
Grade/Class	Name	Remarks
Class I	Clean	Closed, non-infected, and non-inflamed wounds. If the drainage of these wounds is necessary, a closed drainage method is required. In addition, these wounds do not enter the respiratory, digestive, genital, or urinary tract.
Class II	Clean contaminated	Wounds that were made under controlled conditions without unusual contamination. They enter into the respiratory, digestive, genital, and urinary tracts.
Class III	Contaminated	Recent, open wounds result from accidents or a deviation from sterile techniques during operations. These incisions show no obvious signs of acute or purulent inflammation.
Class IV	Dirt-infected	These injuries are the result of the neglect of traumatic wound care. They are older wounds where the tissue is devitalized, and there may be pre-existing clinical infections or even organ perforations. The presence of pathogenic microorganisms worsens the prognosis of the wound.

**Table 2:** Therapeutic effects of different hydrogels with bioactive substances.

Bioactive Compound	Hydrogel Composition	Method of Application	Experimental Subject	Therapeutical Effect/Limitations	Reference
PGE_2_ (Prostaglandin E_2_)	PGE_2_/Chitosan (CS)	Topical (in gel form)	Lab rats	Faster decrease in ROS and pro-inflammatory cytokine (IL-1β) levels; Acceleration of wound healing by decreasing infiltration of inflammatory cells at level of injury; Increased gene expression for angiogenesis-related genes (PLGF, VEGF-A, PDGF-BB, b-FGF, Ang-1, and Ang-2)	[194]
Resveratrol	RSV/(collagen–laminin based dermal matrix)/Hyaluronic acid–Dipalmitoylphosphatidylcholine (HA-DPPC)	Topical (in gel form)	Lab rats (Wistar Albino)	Strong antioxidant activity; Acceleration of wound healing and tissue reconstruction in diabetic wounds	[127]
Chitosan	Quaternized chitosan–Matrigel–polyacrylamide (QCS-M-PAM)	Topical (in gel form)	Lab rats (Sprague Dawley + Kunming)	Antibacterial protection against *Staphylococcus aureus* (*S. aureus*) and *Staphylococcus epidermidis* (*S. epidermidis*)	[336]
Chitosan (CS)	CS–Poly(vinyl alcohol) (PVA) (SNP_E_CHG)	Local (bandage)	Lab rats (Sprague Dawley)	Antimicrobial effect on *S. aureus* and *S. epidermidis*; Controlled release of silver ions (Ag^+^) and epidermal growth factor (EGF); Collagen deposition; Accelerating re-epithelialization process; Efficacy in faster healing of diabetic ulcers	[337]
Chitosan (CS)	CS/Agarose (CAH)	Topical (in gel form)	Lab rats (Wistar)	Improvement in re-epithelialization; Reduction in inflammation secondary to trauma; Antibacterial activity on *S. aureus*	[338]
Nanoparticles (NPs) CeO_2_ (CeO_2_ NPs)	CeO_2_ NPs/Chitosan	Topical (in gel form)	Lab rats	Acceleration of healing process especially of first stages; Exceptional antibacterial and antioxidant properties	[165]
Paeoniflorin (PF)	HA-PF (Hyaluronic acid-PF)	Topical (in gel form)	Lab rats (C57BL/6J)	Polarization of macrophages from M1 to M2; Improving angiogenesis in diabetic wounds; Promotion of collagen deposition and local re-epithelialization	[193]
Collagen (COL)	COL-HA	Topical (in gel form)	Lab rats	Antibacterial effect on *Escherichia coli* (*E. coli*) and *Staphylococcus aureus* (*S. aureus*) objectified by decreasing colony-forming unit (CFU); Promotion of rapid wound healing by accelerating coagulation and cell adhesion processes, vascular cell growth, angiogenesis, and re-epithelialization; Formation of collateral circulation, epithelial layer, and collagen fibers	[244]
Ciprofloxacin (CF)	CF@GH (Graphen-oxide)/SF (Silk fibroin)	Topical (in gel form)	Lab rats (Kunming)	Antibacterial effect on *S. aureus* and *Pseudomonas aeruginosa* (*P. aeruginosa*); Cell growth of fibroblasts, keratinocytes, and endothelial cells; Improvement in cell proliferation and migration corresponding to proliferative phase of wound healing; High healing capacity in case of burns	[246]
Agar	Agar-Fumaric acid (FA)–silver nanoparticles (AgNPs)	Topical (in gel form)	Lab rats (Albino Wistar)	Antibacterial against *E. coli*, *P. aeruginosa*, *S. aureus*; Accelerates healing rate of skin through epithelization, angiogenesis, and decreased lipid peroxidation; Organized collagen deposition	[339]
(Pearl peptides)	(Pearl peptides/selenium-containing block-functionalized PEG)/Polypropylene glycol	Topical (in gel form)	Lab rats	Reduction in cellular oxidative stress; Improving resistance of skin fibroblasts; Promoting angiogenesis for wound healing	[149]
Ferulic acid	(Feruloyl-modified peptide/glycol chitosan)	Topical (in gel form)	Lab rats (Sprague Dawley)	Epithelial and connective tissue regeneration	[340]
Tannic acid	Tannic acid/PVA (Polyvinyl Alcohol)/PEG (Polyethylene Glycol)/chitosan carboxylate/HA	Topical (in gel form)	Rabbits	Acceleration of collagen deposition; Decrease in TNF-α levels; Regulation of VEGF expression with a facilitating effect	[125]
Dopamine	(Dopamine-substituted multidomain peptide)	Topical (in gel form)	Lab rats (BALB/c)	Considerable shortening of inflammatory phase in process of wound healing; Antibacterial effect	[153]
Lactic acid	Lactococcus/(heparin-poloxamer)	Topical (in gel form)	Lab rats	M2 phenotypic transformation of macrophages; VEGF production and protection	[195]
Vitamin E	Chitosan/Alginate/Vitamin E	Topical (in gel form)	Lab rats (Wistar)	Accelerate wound healing process Increase cell proliferation; Promotion of granulation tissue formation	[341]
(Cerium oxide nanoparticles) (CeONPs)	GelMA-DOPA (Dopamine)—AMP (Antimicrobial peptide)—CeONs	Topical (in the form of a spray by spraying)	Lab rats (Sprague Dawley)	Speeding up wound healing process; Promoting tissue remodeling following healing; Antimicrobial, adhesive, degradative, and ROS-neutralizing properties	[342]
5 mg mL^−1^ fibrinogen and 25 U mL^−1^ thrombin with mesenchymal stem cells (MSCs)	Polymerized fibrin gel/5 mg mL^−1^ fibrinogen și 25 U mL^−1^ thrombin with MSCs	Topical (in the form of a spray by spraying)	Lab rats	Reproduction of local culture system for MSCs to facilitate wound healing; Prevention of scarring	[343]
Silver nitrate (AgNO_3_)	PLGA (Poly(lactic-co-glycolic acid))/PEG Polyethylene Glycol)/Ag	Topical (in the form of a spray by spraying)	Pig	Prevention of risk of infection; Controlled release of antimicrobial silver	[344]
Collagen	COL-CS	Injection	Lab rats (Sprague Dawley)	Antibacterial effect on *E. coli* and *S. aureus*; Good hemostatic capacity, accelerate wound healing	[245]
Quaternized chitosan (QCS)	QCS/Curcumin/(benzaldehyde terminated)	Injection	Lab rats (Kunming)	Improve granulation tissue formation; Accelerate healing process; Antioxidant properties; Antibacterial properties on *E. coli* and *S. aureus*	[338]
Hyaluronic acid	Hydrogels (dual-crosslinking) by the separate modification of HA by adamantane and thiols (Ad-HA-SH) or β-cyclodextrin and methacrylates (CD-MeHA)	Injection	Sheep Dorset	Maintenance of myocardial function (contractility) after infarction; Passive stiffness of treated infarct region was higher in test group compared to control group	[250]
Derived from the myocardial matrix	Derived from the myocardial matrix	Injection	Pig	Beneficial effects on heart muscle; reduction in fibrosis after infarction; appearance of new vascularization foci at level of subendocardial area; prevention of left ventricular (LV) remodeling secondary to myocardial infarction (MI)	[251]
Hyaluronic acid	Ad-HA-SH + CD-MeHA	Injection	Sheep Dorset	Maintenance of myocardial wall thickness at 8 weeks compared to other two groups examined; Reduction in ventricular dilatation secondary to MI; Attenuation of reduction in ejection fraction (EF) secondary to MI	[252]
Carboxy betaine (CB)	Poly(carboxybetaine methacrylate) (PCB)-acrylic anhydrid	Injection	Lab rats	Effects on cardiac tissue post-myocardial infarction; Decrease in homocysteine concentration; Cytoprotective action by preventing release into circulation of myocardial cytolysis enzymes (creatine kinase/CK, lactate dehydrogenase/LDH, aspartate aminotransferase/AST); Decreased fibrosis; Promotion of angiogenesis; Preservation of ejection fraction; Physiological structural maintenance of left ventricle by arresting or slowing secondary MI remodeling; Significant efficiency on free radicals	[345]
Chitosan	CS–Dextran–dopamine	Injection	Lab rats (Sprague Dawley)	Antibacterial, adhesive, and angiogenic capacities; Controlled release of AgNPs and deferoxamine in acidic media; Deferoxamine release promoted angiogenesis by increasing expression of hypoxia-inducible factor (HIF-1α) and vascular endothelial growth factor (VEGF)	[346]
Chitosan	CS–Poly(d,l-lactide)-poly(ethylene glycol)-poly(d,l-lactide) (PLEL)	Injection	Lab rats	Antibacterial and adhesive properties; Promotes angiogenesis by regulating gene expression for VEGF and b-FGF; Significantly accelerates wound healing	[347]
Chitosan	CS-Oxidized HA-graft-aniline tetramer (OHA-AT)	Injection	Lab rats (Kunming)	Biodegradable, antibacterial, electroactive, and antioxidant properties; Promotes neovascularization; Accelerated wound healing by increasing granulation tissue thickness, collagen deposition, and angiogenesis	[348]
Pullulan	Pullulan/horseradish peroxidase (HRP)/hydrogen peroxide (H_2_O_2_)	Injection	Lab rats (Sprague Dawley)	Prevention of adhesion of abdominal tissue following surgery	[349]
(Silk fibroin peptide)	(Silk fibroin peptide-grafted hydroxypropryl chitosan)/oxidized microcrystalline cellulose/tetramethylpyrazine	Injection	Lab rats	Strong antioxidant capacity; Accelerating healing process of wounds, preventing formation of scars	[350]
Polyvinyl alcohol (PVA)	PVA/GM-CSF (granulocytic-macrophage colony stimulating factor)/mupirocin	Injection	Lab rats	Reduction in ROS levels; Promotion of angiogenesis and collagen deposition around wound; Percentage increase in M2 macrophages; Acceleration of wound healing in healthy and diabetic mice	[150]
Chitosan	CS/HAox/CT/Fe (Chitosan/oxidized hyaluronic acid/catechol terpolymer/iron)	Subcutaneous implantation	Lab rats (Albino Wistar)	Anti-inflammatory, adhesive, and antioxidant properties; Promotes growth, migration, and proliferation of mesenchymal stem cells; Protects cells from oxidative stress through controlled release of catechol; It regulates activity of pro-inflammatory cytokine IL-1β	[351]

Abbreviations: PGE_2_ = Prostaglandin E_2_; CS = Chitosan; RSV = Resveratrol; HA-DPPC = Dipalmitoylphosphatidylcholine; QCS-M-PAM = Quaternized chitosan–Matrigel–polyacrylamide; PVA = Poly(vinylalcohol); NPs = Nanoparticles; PF = Paeoniflorin; HA = Hyaluronic acid; COL = Collagen; CF = Ciprofloxacin; GH = Graphen; SF = Silk fibroin; FA = Fumaric acid; PEG = Polypropylene glycol; DOPA = Dopamine; AMP = Antimicrobial peptide; MSCs = Mesenchymal stem cells; PLGA = Poly(lactic-co-glycolic acid); QCS = Quaternized chitosan; Ad-HA-SH = Adamantane-Hyaluronic acid-Thiols; CD-MeHA = β-cyclodextrin-Methacrylates-Hyaluronic acid; CB = Carboxy betaine; PCB = Poly(carboxybetaine methacrylate); PLEL = Poly(d,l-lactide)-poly(ethylene glycol)-poly(d,l-lactide); OHA-AT = Oxidized HA-graft-aniline tetramer; HRP = Horseradish peroxidase; GM-CSF = Granulocytic-macrophage colony-stimulating factor; HAox = Oxidized hyaluronic acid; CT = Catechol terpolymer.

**Table 3:** The currently used hydrogels in the market.

Bioactive Compound	Hydrogel Composition	Method of Application	Advantage(s)	Disadvantage(s)	Therapeutical Effect	References
Hydrosorb	Polyurethane– polyurea hydrogel	Topical (in gel form)	Preserve moisture, facilitate vapor and oxygen exchange, and support tissue debridement	The accumulation of fluids can result in skin maceration or susceptibility to infection, causing mechanical weakness	Wounds or cavities with low-to-medium exudate, necrotic wounds	[374,375]
Restylane	Hyaluronic acid	Subcutaneous injection	Nonpermanent properties and the possibility to use hyaluronidase for product removal	Additional treatment is necessary to sustain the initial effect	Restoring facial volume and reducing the appearance of wrinkles	[376]
Suprasorb^®^ X + PHMB Gel	Polyhexamethylene biguanide	Topical (in gel form)	Antimicrobial action, efficient absorption		Antimicrobial action and the absorption of exudate	[377]
Synvisc	Hyaluronic acid	Intra-articular injections	Long-lasting relief, helps enhance joint function and mobility, contributing to an improved quality of life for patients	Possible injection site reactions, may not be suitable for individuals with certain allergies or pre-existing medical conditions	The treatment of osteoarthritis	[378]
Surgifoam	Gelatin	Topical	Hemostatic efficacy, biodegradable	A possible risk of allergic reactions, potential for adhesion formation	Hemostatic agent	[379]
Integra^®^ Flowable Wound Matrix	Collagen, glycosaminoglycans	Topical	The flowable nature of the matrix allows for easy adaptation to irregular wound shapes, contains collagen, which supports tissue regeneration and wound healing, well tolerated	Proper application requires medical expertise, and incorrect application may affect outcomes	Supporting the regeneration of blood vessels and tissue during plastic reconstruction	[380]
Cohera^®^ TissuGlu^®^	Lysine-urethane	Abdominoplasty procedures, (surgical adhesive)	Reduce or eliminate the need for postoperative drains, minimizes seroma formation	Proper application requires surgical expertise, and incorrect application may affect outcomes	Specific to abdominoplasty procedures	[381]
Strataderm	Polydimethylsiloxanes, siloxanes, alkylmethyl silicones	Topical (in gel form)	Strataderm is designed to assist in the management of postoperative scars and other types of scars	Treatment response can vary among individuals, and some scars may respond better than others	Improve scars by maintaining an optimal environment for healing, providing hydration, and protection	[382]

**Table 4:** Hydrogels used in clinical trials on humans and their therapeutical effects and possible advantages and disadvantages.

Bioactive Compound	Name/Commercial Name	Method of Application	Study Design	Therapeutical Effect	Advantages	Disadvantages	References
Nanofibrillar cellulose	FibDex^®^	Dressing	Prospective single-center clinical trial; Up to 6 months follow-up; Human subjects (n = 19; female = 6, male = 13)	Comparable wound healing time to the commonly used copolymer dressing (mean healing time for both dressings was 18.5 days); Complete epithelialization at 6 months; The Patient and Observer Scar Assessment Scale (POSAS) suggested some advances in scar quality with NFC dressing, particularly in terms of thickness and vascularity, compared to the copolymer dressing.	Biocompatibility; Sustainability; No cytotoxic effects; Comparable wound healing; Scar quality improvement; Ease of storage (at room temperature).	Limited availability; Cost-effectiveness not determined yet; Adverse events (partial sliding off the donor site and suspected infection) that may impact patient outcomes; Superficial residual wounds; Limited clinical validation (larger sample sizes and longer-term follow-up are needed).	[383]
Olea europaea leaf extract (OELE)	EHO-85	Gel	Pivotal clinical trial; 8 weeks follow-up; Human subjects (n = 69; female = 48 male = 21)	Superior effect in accelerating wound healing in hard-to-heal ulcers evaluated by wound area reduction (WAR) and healing rate (HR); One in three patients treated with EHO-85 achieved a closure rate of at least 80%, compared to only 9.1% in the VariHesive group; Patients treated with EHO-85 showed a significantly higher average daily reduction in ulcer area; Kaplan–Meier analyses showed that EHO-85 hydrogel treatments were associated with a higher probability of achieving a WAR ≥ 40%, ≥ 60% and ≥ 80% compared to the control group.	Superior wound healing (WAR and HR); Faster ulcer closure (almost 80% compared to the standard hydrogel); High healing rate; Microenvironment modulation (reduces ROS through its antioxidant properties); Easy to apply on wounds.	Limited comparative evidence; Specific patient population; Need for further research.	[384]
Hydrogel enriched with sodium alginate and Vitamins A and E	-	Dressing	Single-blind randomized controlled trial; 12-week follow-up; Human subjects (n = 26, female = 9, male = 17).	No significant difference between the group using the investigated hydrogel and the control group using conventional dressing; Despite the hydrogel’s composition, the study failed to identify a positive effect of the treatment with hydrogel in terms of wound closure or overall wound healing outcomes. Decreased inflammatory infiltrates after 12 weeks of treatment.	Reduced inflammation infiltrates; Ease of application.	Questionable effectiveness of the hydrogel in promoting complete wound healing within the study duration.	[385]
OELE	EHO-85	Dressing	Prospective, parallel-group, randomized, investigator-blinded, multicenter clinical trial Human subjects (n = 213)	Low viscosity at high shear rates; Acceleration in wound healing, compared to those treated with VariHesive; Higher 26% average ulcer closure after 14 days of application; The daily wound healing rate was three times higher in the EHO-85 group after six applications.	Accelerated wound healing; Moist environment; Antioxidant properties; Spreadability (easy to apply on wounds with gentle manual pressure)—it can also enhance patient compliance and facilitate the uniform coverage of the wound area; Ease of application; Biocompatibility.	Low cohesive energy density.	[386]
Hydrogel with urea and papain	-	Dressing	Prospective, single-center, randomized, double-blind and comparative clinical trial; Two formulations: HGP2 (2% papain) (n = 32) and HGP10 (10% papain) (n = 30); Human subjects (n = 62; female = 37, male = 25).	Both HGP2 and HGP10 formulations were effective in wound healing; HGP10 proved to be more efficient, causing no setbacks in the healing process and demonstrating versatility in treating various tissue types in the wound bed.	Easy application; Spreadability; No undesirable effects of complications Storage at room temperature. Autonomy in self-care for patients.	A possible degradation of hydrogel if exposed to higher temperatures during storage or transportation; Density variation; Decrease in enzymatic activity over time—might impact the hydrogel’s efficacy during extended use.	[387]
Triticum vulgare extract + polyhexanide	Fitostimoline^®^	Dressing	Monocentric, two-arm, open-label, randomized, controlled trial; 12 weeks of treatment; Human subjects (n = 40; female = 11, male = 29) with type 2 diabetes mellitus.	Important reduction in the score of erythema and bleeding of the wound; Reduction in signs and symptoms such as pain, itching, and burning; No complete healing of the wound.	Safety; Tolerability; Easy to apply; Moist wound environment.	To be considered regarding the trial: Duration of the trial; Small sample size; Subjective scoring, Potential bias; Limited generalizability (the study focused on diabetic foot ulcers at specific grades and stages).	[388]
Hydrogel/nano-silver based	-	Dressing	Randomized controlled trial; Human subjects (n = 60) with type 2 diabetes mellitus	Lower area of the ulcer; Faster reduction in ulcer size, compared to traditional dressing group; Enhancing re-epithelialization and wound contraction.	Lower cost; Decrease in hospital stay; Safety.	Regarding the trial: Sample size; Need for further research.	[389]

## Data Availability

Not applicable.
